# Supplementary material for: Production of a β-Glucosidase-Rich Cocktail from Talaromyces amestolkiae Using Raw Glycerol: Its Role for Lignocellulose Waste Valorization
Source: J Fungi (Basel). 2021 May 6;7(5):363. doi: 10.3390/jof7050363 (PMC8148544; doi:10.3390/jof7050363)
Supplement: Supplementary file 1 [file jof-07-00363-s001.zip › jof-1202736-supplementary.pdf]

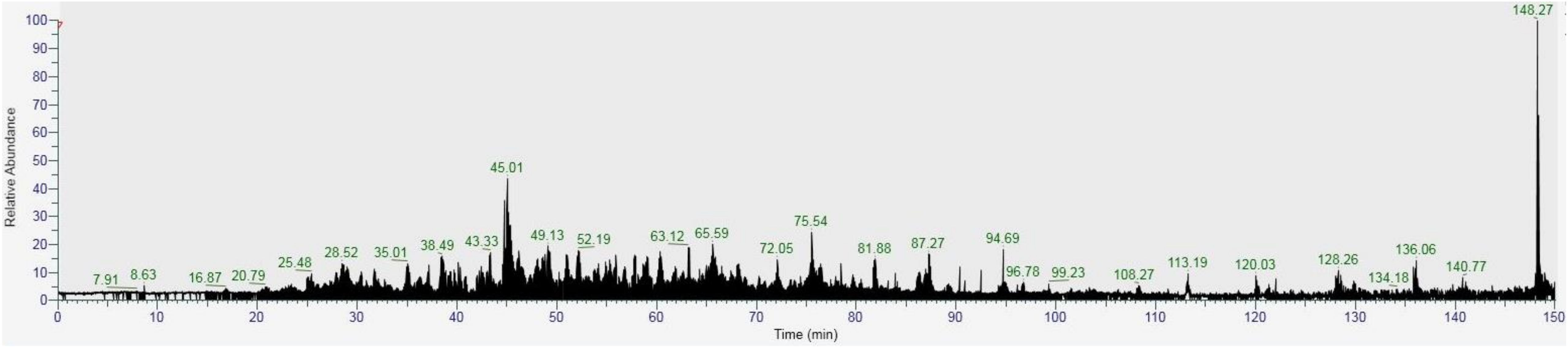

Figure S1. TIC (Total Ion Chromatogram) of *T. amestolkiae* culture supernatant growing in 1% glycerol as carbon source analyzed by nanoHPLC-MS/MS.

Table S1. Detected proteins in the secretome of *T. amestolkiae* cultures, induced with 1% of glycerin as carbon source.

ID	PSM	Predicted protein function	GH family
g377	7.09	beta-glucosidase	GH3
g3995	6.47	glutaminase	
g8295	3.59	alpha-glucosidase	GH31
g2158	3.28	glucoamylase	GH15
g9324	3.18	Exo-beta-1,3-glucanase	GH55
g2140	2.50	glucoamylase	GH15
g5915	2.23	non-reducing end β-L-arabinofuranosidase	GH127
g4076	2.00	hexosaminidase	GH20
g216	1.77	neutral/alkaline nonlysosomal ceramidase	
g9148	1.58	catalase	
g9150	1.54	beta-glucosidase	GH3
g4965	1.52	Thioredoxin reductase	
g6145	1.49	alpha-1,2-mannosidase	GH92
g6554	1.39	mutanase	GH71
g4603	1.36	beta-galactosidase	GH35
g9290	1.33	alpha-glucosidase	GH31
g3307	1.29	hypothetical protein	
g6658	1.20	hypothetical protein	GH125
g3385	1.18	chitinase	GH18
g4355	1.12	manganese peroxidase	GT2
g10182	1.10	endo-1,3-beta-glucosidase	GH17
g3279	1.09	alpha amylase	GH13
g4633	1.08	alpha-1,2-Mannosidase	GH47
g3185	1.07	[N-glycan] α-1,3-mannosidase	GH92
g3077	1.05	Exo-beta-1,3-glucanase	GH55
g608	1.05	1,3-beta-glucanosyltransferase	GH72
g3149	1.03	beta-mannosidase	GH2
g7301	0.96	aminopeptidase	
g3202	0.94	cathepsin	
g1180	0.92	Exo-beta-D-glucosaminidase	GH2
g7148	0.86	Xaa-Pro dipeptidase	
g8384	0.85	β-glucosidase	GH1
g8260	0.81	endo-inulinase precursor	GH32
g6857	0.79	beta-glucosidase	GH3
g10153	0.79	Isoamyl alcohol oxidase, putative	AA7
g5548	0.79	alpha-rhamnosidase	GH78
g2384	0.78	alpha-galactosidase	GH27
g4362	0.77	cathepsin	
g8428	0.77	Glucan endo-1,3-beta-glucosidase	GH55
g2159	0.76	alpha-amylase	GH13
g10298	0.75	hypothetical protein	
g2234	0.73	cellobiohydrolase	GH7
g6228	0.71	amine oxidase	
g4013	0.70	hypothetical protein	
g4461	0.69	alpha-fucosidase	GH95
g571	0.69	hypothetical protein	

g10078	0.69	endo-1,3(4)-beta-glucanase, putative	GH81
g881	0.63	aldose-1-epimerase	
g7294	0.62	hypothetical protein	
g2392	0.61	hypothetical protein	
g9312	0.59	arabinofuranosidase	GH54
g7641	0.58	beta-glucanosyltransferase	GH72
g4318	0.56	peroxidase	AA2
g7147	0.56	leucyl aminopeptidase	
g2989	0.55	Extracellular cell wall glucanase	GH16
g2125	0.55	Alpha-galactosidase	GH27
g456	0.55	glutaminase, putative	
g8806	0.52	arabinan-endo 1,5-alpha-L-arabinase	GH43
g1839	0.51	endo-1,3(4)-beta-glucanase, putative	GH16
g4704	0.51	beta-lactamase	
g8103	0.50	tripeptidyl-peptidase	
g4745	0.50	Glucan endo-1,3-alpha-glucosidase	GH71
g8259	0.48	glucoamylase	GH15
g9337	0.46	alpha-mannosidase	GH92
g3006	0.45	glutathione oxidoreductase	
g7811	0.45	6-phosphogluconolactonase	
g3707	0.45	arabinofuranosidase	GH62
g7845	0.43	peptidase	CE7
g727	0.43	Endo-beta-1,6-galactanase	GH30
g9673	0.42	chitin synthase	
g7487	0.41	conidial pigment biosynthesis oxidase	
g8480	0.41	phosphatase	
g4474	0.40	Glucan endo-1,3-alpha-glucosidase	GH71
g3728	0.38	peptidase	
g1325	0.37	asparaginyl-tRNA synthetase	GH106
g5215	0.36	glucose-6-phosphate isomerase	
g3139	0.36	beta-glucosidase	GH3
g3187	0.36	FAD/FMN-containing isoamyl alcohol oxidase	AA7
g3993	0.36	protease	
g4148	0.36	Tyrosinase	
g8248	0.35	arabinofuranosidase	gh51
g290	0.34	aminotransferase	
g7031	0.33	endonuclease	
g2149	0.33	transcription factor, putative	
g5495	0.33	1,3-beta-glucanosyltransferase	GH72
g5860	0.33	endoglucanase	GH5
g7045	0.33	endo-1,3-beta-glucosidase	GH64
g2283	0.32	xylooligosaccharide oxidase	AA7
g3685	0.32	hypothetical protein	
g5245	0.31	serine protease	
g7406	0.31	beta galactosidase, putative	GH35
g1116	0.30	hypothetical protein	
g3954	0.30	alpha-fucosidase	GH29
g9487	0.30	alpha-L-rhamnosidase	GH78
g2465	0.29	transpeptidase	
g2794	0.29	hexosaminidase	GH20
g4411	0.29	2,3-dihydroxybenzoate decarboxylase	
g9063	0.29	chitinase	GH18
g8017	0.29	formate dehydrogenase	
g5328	0.29	hypothetical protein	

g7486	0.29	hypothetical protein	
g5593	0.28	sexual development protein	
g6147	0.28	fumarylacetoacetate hydrolase Fah	
g7927	0.28	Alpha-L-arabinofuranosidase axhA	GH62
g10375	0.27	hypothetical protein	
g5993	0.27	hypothetical protein	
g395	0.27	hypothetical protein	
g7627	0.26	Vacuolar aminopeptidase 1	
g9490	0.25	hexosaminidase	GH3
g3369	0.25	oxidoreductase	AA3
g466	0.24	chitinase	GH18
g4882	0.24	alpha-1,2-mannosidase, putative	GH92
g2143	0.24	Glucose oxidase	AA3
g7540	0.23	HypA-like protein, putative	
g7482	0.23	Conidial pigment biosynthesis oxidase	AA1
g5789	0.22	arabinofuranosidase	GH54
g6836	0.22	hypothetical protein	
g3001	0.21	glucan endo-1,3-beta-glucosidase precursor	GH64
g1079	0.20	hypothetical protein	
g1266	0.20	Alpha-glucosidase, putative	GH31
g7206	0.20	glucan 1,3-beta-glucosidase	GH5
g10174	0.19	dienelactone hydrolase	
g4950	0.18	α-D-xyloside xylohydrolase	GH31
g6249	0.18	Mannosyl phosphorylinositol ceramide synthase	GT32
g1197	0.17	peptidase	
g3578	0.17	hypothetical protein	
g3952	0.17	2,3-dihydroxybenzoic acid decarboxylase	
g9061	0.17	alpha-1,3-glucanase	GH71
g8069	0.14	actin Act1	
g2575	0.13	protease	
g4734	0.13	hypothetical protein	
g4878	0.11	aminotransferase	
g5497	0.11	aldehyde dehydrogenase	
g5707	0.11	endoglucanase	GH6
g7783	0.11	Putative epoxide hydrolase	
g3373	0.09	beta-xylosidase	GH39
g5712	0.09	Mannan endo-1,6-alpha-mannosidase DCW1	GH76
g6753	0.09	beta-glucosidase	GH3
g6784	0.09	lipase	
g6921	0.09	arabinofuranosidase	GH43
g9076	0.09	feruloyl esterase	GH3

A2	Sequence	# PSMs	# Proteins	# Protein Groups	Protein Group Accessions
s detected in the LC-MS/MS analysis c		# PSMs			
High	VAFTNAAAGTADGDSVGTSGVDLL	1	1	1	g1197.t1
High	ATAMIAYANYLIDNSQSSTAK	1	1	1	g2158.t1
High	DYGAAGDGVTDDTAAINSAISGGG	3	1	1	g9324.t1
High	NVKDYGAAGDGVTDDTAAINSAIS	6	1	1	g9324.t1
High	NWEGFAPDPVLTGQmmASTIEGm	9	1	1	g377.t1
High	EYINVETVIQGISNPSGSLADGTGL	9	1	1	g2158.t1
High	QTQLAFSEINDQTEYGNWWYATT	13	1	1	g3995.t1
High	TDASQEQTDVQSFSDmEPIADGFR	1	1	1	g4318.t1
High	ATAGFTGLGLIDGDQYQSSGNQGV	4	1	1	g9324.t1
High	GDGSTDDTAAIQSALNSAAASGSIA	2	1	1	g9324.t1
High	TQVPINFASWTLDTYGNEYYYAGE	3	1	1	g377.t1
High	YDVmVNPDAVDGLANFNWGGNG	1	1	1	g4076.t1
High	SGKDANTVLASINTFDPQATcDDV	7	1	1	g2140.t1
High	NVAESDSQITGDLILAGSAcNTYGE	5	1	1	g8295.t1
High	SSDNGIYAIGDANNDGATNVPHAm	1	1	1	g4965.t1
High	DVYSSAAVGTYASGSTAFTAIISAV	4	1	1	g2140.t1
High	SQVLDSVNYVLSHQQADGWLGPE	11	1	1	g5915.t1
High	AFESTSGSISDLYNmVNTVK	3	1	1	g2159.t1
High	ISVFGNDAADLSGGPYDPSNTNGV	2	1	1	g9150.t1
High	NWEGFAPDPVLTGQMmASTIEGm	4	1	1	g377.t1
High	DANSLLGTIHTFDPEAAcDDTTFQF	1	1	1	g8259.t1
High	LASQTASDGYPWASLcQTDTSGAY	2	1	1	g3001.t1
High	QVIAPDTPYVATAGPNQLYFIDTR	2	1	1	g10375.t1
High	TLWNTGQAFLPTHSNLYGSHPIYIE	2	1	1	g8295.t1
High	AFLVTGVTGPcVGNIAAIPR	2	1	1	g9150.t1
High	LGANVDGSWTSFGQQGPImPYDT	2	1	1	g6228.t1
High	ATAmIAYANYLIDNSQSSTAK	4	1	1	g2158.t1
High	DFVDTVTGDRFQIVGVDYQPGGSS	3	1	1	g5495.t1
High	YGLGADQVVEWEVIDGTGK	1	1	1	g3187.t1
High	YDNTLAFFSGNEVINDGPSSK	2	1	1	g608.t1
High	AIGESSDPEQAASQAGSDAAAALG	2	1	1	g9490.t1
High	TmDNNAASDALATTcSVAIDK	2	1	1	g4013.t1
High	NHNLDGFDmDVEENVSESVLLR	1	1	1	g9063.t1
High	NPAGGSGEVVYDASAGVYAR	1	1	1	g10078.t1
High	RISVFGNDAADLSGGPYDPSNTNG	1	1	1	g9150.t1
High	FADGFIAGFSQSSVGDTSPNVLGAY	3	1	1	g216.t1
High	DGAGQIFIPLNTAAYSPNTLNNGSP	2	1	1	g9148.t1
High	YVPDGAQDGSPQPVNPAGGAPGGI	16	1	1	g377.t1
High	GAVAPLWSEQVDDTVISGK	6	1	1	g4076.t1
High	GYTQGGSNADVLIAEAYLK	1	1	1	g3185.t1
High	SWDWPNPIIASAGELGFPDALGSS	2	1	1	g1180.t1
High	ADGSLIGTFVDTIGGVGALDQSPN	2	1	1	g466.t1
High	NPLNYFAETEQVmFQPGHIVR	2	1	1	g9148.t1
High	ITNANPGSSYTYGDNSGSADSIK	2	1	1	g2989.t1
High	DDTLTFYLNPGPALVEPSSSNTADV	1	1	1	g7045.t1
High	SVYSLDSGIAEGVAVAVGR	6	1	1	g2140.t1
High	FATLVYTVHELANNPDLAASTLDGL	1	1	1	g10078.t1
High	LQDGSTNNDPAQDLEFDLFR	4	1	1	g4965.t1
High	DSALENDLITSSYAQGmANR	2	1	1	g571.t1
High	DTVNVLIADAGQGAIYK	2	1	1	g7486.t1
High	LAPAAAGSEcGVYGNDPDQGQTAV	2	1	1	g4745.t1
High	VQSDASAISDNYATLATLALR	13	1	1	g3995.t1
High	LFGGGHmVPYDQPEASLEFFNR	5	1	1	g3202.t1
High	HmHDVIGNDGTVPAVFR	5	1	1	g4965.t1
High	TYDYIVIGGGSGGSGSAR	1	1	1	g3006.t1

High	YLGGLLAGYDLLSGPAANLVSDK	7	1	1	g4633.t1
High	AcQNAETEIPEPTSNFADIQSAAEQ	1	1	1	g4882.t1
High	LVmAPLFENQEAGQYPNR	5	1	1	g3995.t1
High	YLPLLSGNEGELSAAEGPIR	1	1	1	g1325.t1
High	QTTLGTETLSLTGEGNPLNR	5	1	1	g6658.t1
High	ALWVTETGWPVSGDKENLAVASTD	2	1	1	g10182.t1
High	TSLPSNPNIYGLGEDSDSFK	1	1	1	g8295.t1
High	VYTVASDcGVSTAcINDSWQ	2	1	1	g2158.t1
High	IcATGGVGSLLDDPEDAQFSPEEIK	1	1	1	g3307.t1
High	VDATAAGPIGVPLPQNSQYTFIQQ	1	1	1	g4355.t1
High	NTALTGScTILSGSNPVAYGYLGcY	1	1	1	g4355.t1
High	SVYSLNSGIAASSAVAVGR	8	1	1	g2158.t1
High	VPTSAGTLTLPQLGGSLTLNGR	1	1	1	g4603.t1
High	YmGPLPYGNLGVGEYQYGGR	2	1	1	g10153.t1
High	RDVSNWDTVSQNWVVSNYTK	2	1	1	g377.t1
High	NWEGFSPDPYLTGISFAETIK	2	1	1	g9150.t1
High	NLGYIPTDDFDPYGQGPLTR	2	1	1	g3185.t1
High	SSNLVNSATWAGYDLQVTK	2	1	1	g6228.t1
High	TVEYSLNDFALSQVAAGIQPADK	2	1	1	g9337.t1
High	GLTDGIDWDTAYAAVQK	3	1	1	g6145.t1
High	TRFNPIYPAScPFVTSVGGTYR	1	1	1	g8103.t1
High	SGAVTFETNPEVSSSSITSSWSK	2	1	1	g3385.t1
High	AGATDISQITPPNSAPVGALWYR	3	1	1	g4474.t1
High	VYTLNTVGVTNmIEVDGTSEAK	8	1	1	g9324.t1
High	ELAYAVEAGmSPLEAIEASTAR	1	1	1	g3307.t1
High	AGmVADSGALASAGYQK	1	1	1	g7301.t1
High	TQmSQGAVAPDLLYFAPK	2	1	1	g3707.t1
High	ATAVSQAINDNLWDASVGAYVDEr	2	1	1	g5548.t1
High	DFGAKGDGSTDDTDAINSAISSSDI	1	1	1	g3077.t1
High	SSGAKGDGSTDDTAAIQSALNSAA	2	1	1	g9324.t1
High	FVAVFGEDAGSNPDGVNGcSDR	2	1	1	g377.t1
High	DVSNWDTVSQNWVVSNYTK	3	1	1	g377.t1
High	DMFYSDLANWINVTPTNR	4	1	1	g3995.t1
High	GGPIILYQPENEYSGWTTGYSDDG	2	1	1	g4603.t1
High	YAATELEDLEIVDNK	2	1	1	g3202.t1
High	VYTLNTVGVTNMIEVDGTSEAK	2	1	1	g9324.t1
High	IVNYGGDSSTTVTVLVPGK	2	1	1	g8248.t1
High	FISDASDQGYGFVSDFR	1	1	1	g1325.t1
High	AASHmcLTATDSVYTNSLPVQAcE	1	1	1	g4013.t1
High	SADNLWYVTLNLPSGTAFEYK	2	1	1	g2158.t1
High	GGVLEITTGASESAWGTK	2	1	1	g3185.t1
High	VSVEDVQGGFFQDLNALQGQR	3	1	1	g10298.t1
High	ELANGPEQYWGPNLETLIAIK	2	1	1	g4895.t1
High	GKGVDVQLGPVAGPLGR	3	1	1	g377.t1
High	DVVIIGGGASGAYAGVR	1	1	1	g10298.t1
High	STLLIESNTPESDDPTK	1	1	1	g7045.t1
High	SIYTINSGIAEGEGVAIGR	1	1	1	g8259.t1
High	TLVISEDGSLVAVGNQK	1	1	1	g7811.t1
High	NSPTGIAASAGIGKNPDDIVNDIK	2	1	1	g10182.t1
High	ETSESSFLNYALNAGLPLTVYK	4	1	1	g3369.t1
High	QVLGATSFAGTAENPLLFLK	2	1	1	g456.t1
High	GDQSFAPDDSVAFLDGSSDASGTL	2	1	1	g7811.t1
High	TVEAQSGSAYVIYSDIR	2	1	1	g2234.t1
High	ELEAAFIGTcIAQGcR	2	1	1	g7148.t1
High	DSSVNPLTYTDGPPSAAR	3	1	1	g571.t1
High	TLIDNFIAGTSDLESTIK	5	1	1	g2158.t1
High	GDWESWAASLASESTR	7	1	1	g3995.t1
High	LSLGAGEEAQWTATLTR	10	1	1	g377.t1
High	IAQYASLPAQLMVDNK	2	1	1	g6554.t1

High	QSAPYAPLLVTGTETSNDGALSAPC	1	1	1	g8806.t1
High	AVGAEVDSFDScNFDINR	2	1	1	g3202.t1
High	YDNcYSDAATNYPNVNYAPSTSPEA	1	1	1	g2125.t1
High	AIGAQTSYQEcPNGPYNK	2	1	1	g4362.t1
High	ALFASSITTASDVVTLQ	1	1	1	g8806.t1
High	VQSDFESEFKTAAGLVGAEDFTSAF	1	1	1	g10182.t1
High	VGVSFISVDQAcSNGEKEIPDFDFD	1	1	1	g3185.t1
High	SATNERELEAAFIGTcIAQGcR	1	1	1	g7148.t1
High	TVEYAYNDYcVGVVGK	1	1	1	g4882.t1
High	GDGSTDDTDAINSAISSSDR	3	1	1	g3077.t1
High	HSSNQLLLNANDGTK	2	1	1	g5789.t1
High	TLHITATGLDYNEGSYYVQSVR	1	1	1	g9337.t1
High	IGLGNSGcYSFESVDTPGSYIR	2	1	1	g5789.t1
High	DLTWSYAALLTANNR	2	1	1	g2140.t1
High	SAGAKGDGTTDDTAAIQK	2	1	1	g3077.t1
High	ADEVTTFPLLVQAIR	5	1	1	g3385.t1
High	IGYIGPGGTAVTTISAASAGTK	2	1	1	g2125.t1
High	VFVSSFAGDGVDVAALR	5	1	1	g6554.t1
High	HAQDGNAIAVIAGIANSSQAASALK	1	1	1	g5548.t1
High	VAGVEVPQLYVSLGGPSDAPK	3	1	1	g377.t1
High	LTYSIDmALDPTQSVSASFTAAGK	1	1	1	g5860.t1
High	GYTQGGSNADNVIADAYVK	2	1	1	g6145.t1
High	TLEYAYNDFAISQIAR	1	1	1	g6145.t1
High	GSDLLVIDTGDRVEGNGLYDASNP	1	1	1	g8480.t1
High	DANTVLASINTFDPQATcDDVTFQF	9	1	1	g2140.t1
High	VSLNLGNSAGSTSSLPTNQR	2	1	1	g4461.t1
High	SPLLLELSGIGNPDILK	2	1	1	g2143.t1
High	DLGNDVLYNSTVLQSLR	2	1	1	g10298.t1
High	NADLIAFNQDPVIGKPALPYK	4	1	1	g2384.t1
High	TVDTTKPFTVVTQFVTSDGTNTGT	4	1	1	g2234.t1
High	TVQTLSDGVNDDESYSAGAFTR	2	1	1	g3185.t1
High	GAPTLIGGTSAAAPVFAAILTR	1	1	1	g3993.t1
High	YTFTPAAGVITLAEADIR	4	1	1	g1839.t1
High	IIGEGWAVLSGAGGAFTDPYNPR	2	1	1	g8428.t1
High	YFTTDPASDcATNPIGcATVQLEDA	3	1	1	g3385.t1
High	IGQLIVIVSPSEVTTmSGR	3	1	1	g216.t1
High	GYPDVAAIGDNVLIYNR	2	1	1	g3993.t1
High	FWGNAFmDDDSLGSGTSDR	2	1	1	g5548.t1
High	LIIAAVmNSYGSNPPTNTWK	4	1	1	g8260.t1
High	IAQYASLPAQLmVDNK	2	1	1	g6554.t1
High	TQTLQVAAPTEDLGLDGPVSQLPGI	3	1	1	g4013.t1
High	DIGLFQDDDGSAYLLTEDR	1	1	1	g5620.t1
High	QINDVATDTQGFVGYSTSK	1	1	1	g6784.t1
High	VTGNIEQLPVAISLLGAPNR	2	1	1	g7294.t1
High	AIQDSWPVFAHAFDLGR	11	1	1	g3995.t1
High	TNPDQTQTIDAcNLQFLYQGR	1	1	1	g7927.t1
High	RYTEDQYDVWSGPLSGQR	1	1	1	g2125.t1
High	ADGLNKFVFNTEAHPFK	2	1	1	g4633.t1
High	DTGAPLSASALTWSYAALLTAAAR	9	1	1	g2158.t1
High	VLNATAYLFPNYIK	1	1	1	g377.t1
High	KAVLNQGVFEHPTYGK	2	1	1	g6658.t1
High	IYDENEQVYQIPK	3	1	1	g8295.t1
High	EALWTSDYSTTAQLYTHTK	3	1	1	g3279.t1
High	ADPEVSYNDVYLQNVFATVEmFAK	2	1	1	g608.t1
High	GVTISDAIEAGALTAFGSDSDR	2	1	1	g9490.t1
High	TAAVSAAcEAGFTSLHITSSAK	3	1	1	g9324.t1
High	TGLGNSAcYSFESVDTPGSFIR	3	1	1	g9312.t1
High	KPcEDSSLcYADLEFVSTYLNQAEVI	1	1	1	g3202.t1
High	TPLVLSEFGFPVYAESTR	3	1	1	g8384.t1

High	SNNWADWYVDGVYIK	4	1	1	g5915.t1
High	DIDNAITALQESWAEVK	3	1	1	g290.t1
High	SDPIENFSAWcLATQR	2	1	1	g3149.t1
High	IcATGGVGSLLDDPEDAQFSPEEIK/	3	1	1	g3307.t1
High	YITHTGSTVNTQVVTSSDDLTTR	1	1	1	g9312.t1
High	TYPLEGTmVFFPQYAPYTDPIR	3	1	1	g4355.t1
High	LRTSLPSNPNIYGLGEDSDSFKR	4	1	1	g8295.t1
High	DEYGPIYAAAGPEYGVGK	1	1	1	g6857.t1
High	TWmPTDPYIYGLGEDVDSFR	2	1	1	g9290.t1
High	FNPIYPAScPFVTSVGGTYR	2	1	1	g8103.t1
High	WTSSGSLANPASGYLSLK	1	1	1	g3707.t1
High	FLIGDADSALDEIHR	2	1	1	g5548.t1
High	EAQPTFDSNGQFVTNVYTWQR	2	1	1	g6658.t1
High	DVEISQTGPVSLAPIR	2	1	1	g1180.t1
High	GKGAHVALGPVAGPLGR	1	1	1	g9150.t1
High	LVMAPLFENQEAGQYPNR	6	1	1	g3995.t1
High	cQSmIQScYDSESAWTcVPASIYcN	1	1	1	g3202.t1
High	EAGLGDEEAVELFSSK	1	1	1	g5328.t1
High	YGTGANTYDTDIQEFPEEFR	3	1	1	g2392.t1
High	YIEPQYADTFEFYNGER	2	1	1	g7487.t1
High	ELPAVDITNPTTDVSEYVVGGK	2	1	1	g1325.t1
High	AEGIFDLQPFFNAAK	2	1	1	g4603.t1
High	NHADISANYQEFYR	4	1	1	g8295.t1
High	DmFYSDLANWINVTPTNR	10	1	1	g3995.t1
High	VGLSFISEDQAcSNAEK	1	1	1	g6145.t1
High	TGLDANSILGVIETFDPEASScDDA	6	1	1	g2158.t1
High	DYLTAAGSILTVEAR	1	1	1	g7797.t1
High	SAFRPSDDATIFGYFVPANAmmSV	4	1	1	g6658.t1
High	EAIGDDKVLSIATPGLQR	2	1	1	g3385.t1
High	TYSNVVILSDLTPATTYYYK	2	1	1	g10048.t1
High	AGGAQGGNPALWDTVFTVDLK	1	1	1	g3139.t1
High	FESISGNEADVGDYVIQYLQAR	2	1	1	g3728.t1
High	SNNNNVNDEFQSIDGFPAPNNQQI	1	1	1	g5593.t1
High	FQFPTGDSSASPLILLDLTDLSDSR	1	1	1	g6145.t1
High	TGDVKPEEDETVYPVFLALR	2	1	1	g7301.t1
High	ADEINQIFDAISYSK	1	1	1	g7301.t1
High	QVIVVNKDQPNEFGEYPGYR	1	1	1	g6228.t1
High	QPIDTITSWIADNGFNcVR	1	1	1	g5860.t1
High	NDmGYHNIEANSILVYTLK	1	1	1	g9487.t1
High	TPSSWESETITWYmDGK	3	1	1	g1839.t1
High	SGGScTTNAGSITLDANWR	1	1	1	g2234.t1
High	SSADQTKPVDPcNLQLLYQGR	1	1	1	g3707.t1
High	SILDPPSGQQDSSSQQSDLVFEYTK	8	1	1	g8295.t1
High	SITWGTSGPISVPQNLNVQLPR	2	1	1	g4076.t1
High	QDLDTVSVDITNAEYAFEHGVNIG(	6	1	1	g5915.t1
High	AYIAPASPWFSTHFGPEVSYSK	5	1	1	g6554.t1
High	HSDYTSLDTTSYTLK	2	1	1	g4603.t1
High	EIFSPADAPVVNLATYQFGTL	5	1	1	g881.t1
High	QYAEVVGAPAEQAYWTYGFHQcK	1	1	1	g9290.t1
High	QTGYDIFcSDSILIQGK	1	1	1	g7487.t1
High	YAGTcDPDGcDFNPYR	1	1	1	g2234.t1
High	IFSEDATFcPQSGLNGQGNSFR	2	1	1	g9312.t1
High	ANSGATWTNDISSGDLIR	1	1	1	g7927.t1
High	IGPEVFAWDEAGVPADQEAFYK	4	1	1	g4633.t1
High	IGNDISSSWSSIYR	1	1	1	g2125.t1
High	TFTTAETTTNANSAK	1	1	1	g5215.t1
High	DAEVTPYNTFDPTDFTASTK	2	1	1	g9337.t1
High	SDcADTSLLGNFIENHDNPR	3	1	1	g3279.t1
High	ADGTYVmYFSAAAASDSSK	2	1	1	g8806.t1

High	FSYEQIHPIEPGSTTLVAR	1	1	1	g4704.t1
High	RSTGTYVVcDGVSDcVK	1	1	1	g3307.t1
High	EVSYASVFPAGVSVAATWDR	1	1	1	g9150.t1
High	TRDTVNVLIADAGQGAIYK	2	1	1	g7486.t1
High	QLLVAIEPTTQNmAPFDLDDNEKN	2	1	1	g10298.t1
High	DSmISALLLEPILSK	2	1	1	g395.t1
High	DISNWDVVLQDWVVTR	3	1	1	g6857.t1
High	TDADWNLIYQWER	1	1	1	g216.t1
High	NAAGTVGLAQVPAGSVTDK	1	1	1	g9526.t1
High	GIAYQLSDNDPLVNEAQcK	1	1	1	g886.t1
High	GTLLLSGGNVQPSLSADSYYTTSPc	1	1	1	g3001.t1
High	YTGDADFVAEQWNR	2	1	1	g9487.t1
High	VGVSFISVDQAcSNGEK	1	1	1	g3185.t1
High	ASGKPVEEVmDVWTK	1	1	1	g7301.t1
High	LHVKIYDENEQVYQIPK	1	1	1	g8295.t1
High	QPDWQETFFGSNYDALLK	3	1	1	g10153.t1
High	IDGNSLDGDGTTHSTPImLQQmEA	3	1	1	g8806.t1
High	GLSANQFIVFLER	1	1	1	g7147.t1
High	ITNEFVPQLEAVTPGSGcYQNEANF	3	1	1	g10153.t1
High	FGKPVGAVGGASAALSAVDISTDR	1	1	1	g9148.t1
High	AVEFVSQLTLAEK	3	1	1	g377.t1
High	GRGPYFLENDAGTK	3	1	1	g216.t1
High	FSYTESPFSFTVQR	1	1	1	g9290.t1
High	VGGSAGTGLQSDTcSK	1	1	1	g3077.t1
High	AVPQVYLSFPSSQTNISDDGLPTPV	2	1	1	g9150.t1
High	WSLTEGYAPNPPLLADR	3	1	1	g5915.t1
High	NIYIQSATLNGQPYTK	1	1	1	g6145.t1
High	DFAFNALNQNFIVPGScPNADQIDA	2	1	1	g5593.t1
High	TYWDEVGcGLLFDK	1	1	1	g10182.t1
High	VIDSGYDYWYLDcGR	1	1	1	g2794.t1
High	ASLVWEEAQAVSGK	1	1	1	g9148.t1
High	GSTNmPAEVFLPGGVYTLGSTLTLF	2	1	1	g8428.t1
High	LAAFAATEEPIEcVDYQAPWNPR	1	1	1	g7294.t1
High	LASGTTNQVQEYLGIPYAQPPVNEL	1	1	1	g10240.t1
High	DSIQWLIDGSVVR	3	1	1	g2989.t1
High	QKYDPDYFFYALK	1	1	1	g10153.t1
High	TALNSNPTGIAAAIR	2	1	1	g10153.t1
High	DTTTSINAISLAILK	1	1	1	g2125.t1
High	LSYYcENGPQVDTLILSFVTR	2	1	1	g9455.t1
High	FLTTEQALADVAYFAR	1	1	1	g5245.t1
High	AFIHEPSAEQIAcQAPK	4	1	1	g216.t1
High	NYALQWDNGYTTYK	1	1	1	g2159.t1
High	AFSAAITPcSFSPQGPTR	1	1	1	g4355.t1
High	DTDYNTAFPAGVNVAATWDLDLAY	1	1	1	g377.t1
High	DTGALSLPANLQGIWNEDFSPSWG	2	1	1	g4461.t1
High	SPLIIGTDLSTLPETHLSILK	4	1	1	g2384.t1
High	ALVGNDIYNAVLPAWR	2	1	1	g3187.t1
High	DAAILQYLGVNSIR	1	1	1	g5495.t1
High	QLLVAIEPTTQNmAPFDLDDNEK	1	1	1	g10298.t1
High	IGFNGLcLQDGPLAIR	2	1	1	g9150.t1
High	EYLVANGVQAAPLVPK	1	1	1	g4076.t1
High	TLTAAEAGDYFPQTPMR	3	1	1	g2989.t1
High	ELGLNLVPESVYEmQSNFYPTVNGI	8	1	1	g3995.t1
High	TVAALVNWADEAR	1	1	1	g2125.t1
High	SSQTFVLSGLSASTVTAK	1	1	1	g9236.t1
High	NGVIQGLANLGGEYAR	2	1	1	g216.t1
High	IGSITITSTSLAFFK	1	1	1	g2140.t1
High	VLDSATSDQIVYFDHGAYLITSTIK	2	1	1	g3077.t1
High	NNPGNDVQLVTLLYNFGR	2	1	1	g4461.t1

High	IGGFTGSDLQVANcPK	3	1	1	g9324.t1
High	mSTYPQmTNDAYSPSQTFSHDDIK	3	1	1	g4076.t1
High	VHTYLYGPGSIFVAHGADEAITVR	1	1	1	g3728.t1
High	AAFITNFPDDQQSTLPR	3	1	1	g4965.t1
High	THDFEILVAPDVDGK	4	1	1	g8295.t1
High	SYTVPTGcAGLTATVDDTWR	2	1	1	g3279.t1
High	EDDDFENPGDPTPENPSGIALLR	1	1	1	g6836.t1
High	QEGTFAAVEFLNPK	2	1	1	g216.t1
High	LIVEEGLKNPSIFPPTSYQK	3	1	1	g3307.t1
High	YTPADGTLAEQFQR	5	1	1	g2158.t1
High	NDGSAGEEIAVPANK	3	1	1	g3685.t1
High	GLTDGIDWDTAYAAVQKDAEEEPY	1	1	1	g6145.t1
High	TVDTTKPFTVVTQFVTSDGTNTGT	3	1	1	g2234.t1
High	TAAGLVGAEDFTSAR	2	1	1	g10182.t1
High	ILNFREYLVANGVQAAPLVPK	1	1	1	g4076.t1
High	LFSEDATFcPmAGLNGQGNSIR	1	1	1	g5789.t1
High	QYAGVVGLPAQQSYWTYGFHQcK	2	1	1	g8295.t1
High	EVFLTGGLPPLINGPDPIYEK	3	1	1	g7845.t1
High	TGVVTGGSVSDDFVK	1	1	1	g9148.t1
High	TLTAAEAGDYFPQTPmR	2	1	1	g2989.t1
High	ITGNLGGEDYVDLTR	1	1	1	g4603.t1
High	TTDLSGTISNVQLVYVDR	2	1	1	g4355.t1
High	GNYDGASLcESTYSSTRPPVPYGK	1	1	1	g9673.t1
High	DLLNPPYPLGTVDGIIYGGTIFTDR	3	1	1	g9290.t1
High	GVDVQLGPVAGPLGR	5	1	1	g377.t1
High	MTVTQDQSLSAGEYSR	1	1	1	g5548.t1
High	ASTNmITSSDGTSLALQEDNTSSFA	1	1	1	g3077.t1
High	LVGmFDDEEVGSLLR	1	1	1	g7627.t1
High	NYANHIGTTIAANQITK	1	1	1	g4603.t1
High	QTLEDALYFEDGYK	1	1	1	g9673.t1
High	SQmPFLTLIDPETVTK	3	1	1	g6145.t1
High	LQmAQFmNcGTDDER	2	1	1	g608.t1
High	LPVSSLYIDIFQK	2	1	1	g4603.t1
High	TYNDVTQYWVDHAFPIFK	3	1	1	g4076.t1
High	QLTVGGWSGVSTEGIR	1	1	1	g4355.t1
High	SSVSTQTSTVTVASNR	1	1	1	g2125.t1
High	TQTEILEGNYGGGTAmR	1	1	1	g5374.t1
High	DSVNQVmNYQTLAK	2	1	1	g6658.t1
High	VANAVDFSGHAEFSAK	3	1	1	g4362.t1
High	DALPDYAITYAPLSYLYSGEK	4	1	1	g10314.t1
High	ALSQHVHSVVGGNGFASTmDFAQT	1	1	1	g2392.t1
High	AANDELASAVAANPTR	1	1	1	g3952.t1
High	YGIVTYYNALYVLALQDAAK	2	1	1	g5548.t1
High	SVVEYPLPAAAETHEIVAVSDNLLLI	2	1	1	g3685.t1
High	SHPSILLWYTGDEPDGK	1	1	1	g5993.t1
High	QDAPHPHEVFLDPTNSYILSPDLGA	3	1	1	g7811.t1
High	FSGNPIISAALEAPHDVTGGLESR	1	1	1	g8260.t1
High	GYTYESGIDVDVR	4	1	1	g3995.t1
High	ELETTLLAGFTTIR	2	1	1	g3307.t1
High	GYDIPLYFTFGNSSGSSYR	1	1	1	g4603.t1
High	SSESALTQGSAGAVLR	1	1	1	g4733.t1
High	TTFDLWEETQGSSFFTTAAQHR	4	1	1	g2158.t1
High	SPYAYLANPEDERAQYEYDTDK	1	1	1	g216.t1
High	DLGGYLALDDLK	1	1	1	g2465.t1
High	SKPQYETYGTGSVVSVR	3	1	1	g9324.t1
High	SYTVPSGcGVSTATESDTWR	3	1	1	g2140.t1
High	SGADDYVDIVTLSAR	1	1	1	g456.t1
High	WYTALDTVLQTLDK	3	1	1	g6658.t1
High	NYPNTAGFFSGNEVINEDSVELVPN	2	1	1	g5495.t1

High	TSLPSNPNIYGLGEDSDSFKR	1	1	1	g8295.t1
High	SHmAFPAIGDYNTGIcPSSHPR	2	1	1	g4013.t1
High	STGTYVVcDGVSDcVK	2	1	1	g3307.t1
High	EVGGIAGEIWPAIK	1	1	1	g3307.t1
High	WSDYDAPQPIAIINVAAEK	1	1	1	g2283.t1
High	SKPQYETVAASSFVSVK	2	1	1	g3077.t1
High	LLPDTEPQDYVTNENYYLYK	3	1	1	g8384.t1
High	FLEHPDQFADAFAR	1	1	1	g4318.t1
High	NHHYPPGGLSTDTTLSAK	1	1	1	g3149.t1
High	GQAIIAWDVSSSR	1	1	1	g4603.t1
High	YSYmIHAEDADWQTK	2	1	1	g4633.t1
High	AVLNQGVFEHPTYGK	4	1	1	g6658.t1
High	DAGNSVSNDPSFPFSR	2	1	1	g10078.t1
High	EGYDADVIAVASNPLDDINILGEPE	1	1	1	g3307.t1
High	AGAQYGVGYcDSQcPR	2	1	1	g2234.t1
High	FKQNVPIPSYLFAVASGDLTEAPVG	1	1	1	g7147.t1
High	IcEAYGAPTSGNAAGK	2	1	1	g7482.t1
High	GFNYGSTFNDNSVK	1	1	1	g10182.t1
High	ALAPNSGAYmNEADFR	1	1	1	g3187.t1
High	SGVQFVPSNFLPSDPSR	1	1	1	g6228.t1
High	FEDSTcNGQTELcR	1	1	1	g216.t1
High	LDHLYGGLSVIK	1	1	1	g5993.t1
High	VANSPQNLAWYSISTK	1	1	1	g8428.t1
High	GVDIQLGPVSGPLGR	1	1	1	g6857.t1
High	VTFDSQISTTQVR	1	1	1	g5548.t1
High	YNAPGPGASLPNPIWTLNAPPGSIS	5	1	1	g5915.t1
High	DFDGAmSWENVPASDGR	1	1	1	g8260.t1
High	TPSSWESETITWYMDGK	1	1	1	g1839.t1
High	IPNTTPLTDFEVPVEAIER	3	1	1	g7031.t1
High	SLGFANcLIDDYPEIPSR	2	1	1	g6836.t1
High	SQMPFLTLIDPETVTK	3	1	1	g6145.t1
High	DRDPYVGDIAVSGK	2	1	1	g5548.t1
High	NVmDDANIPSLLALPYLGFLDVDDE	2	1	1	g6658.t1
High	AAALAELVWSGNKDPK	4	1	1	g4076.t1
High	IVLGTGmKDILPITPGVVEGWGK	1	1	1	g4965.t1
High	DGAWLHQQIFDAVVK	2	1	1	g9673.t1
High	GIDFTEDPLLQGR	2	1	1	g9148.t1
High	LPEGVTAPDILPGLLK	2	1	1	g290.t1
High	SDNQGWGDYGFLSR	1	1	1	g5548.t1
High	FLPLGGGYSPDmFmR	1	1	1	g1180.t1
High	LLQAFYAQFPEYESR	2	1	1	g4362.t1
High	SNIFQTISWLDSSK	1	1	1	g3954.t1
High	QIPVGYSAADIDTNR	2	1	1	g608.t1
High	EPSNDPYPPSTYSTYLSNSR	3	1	1	g4362.t1
High	VSIADLIVLAGSAAVEK	2	1	1	g4318.t1
High	HYGYDVQDNIK	1	1	1	g3006.t1
High	LPGGNNLEGIASPYR	1	1	1	g8248.t1
High	YDTQSSGLASGR	1	1	1	g8480.t1
High	VIEVTSNDGSETK	2	1	1	g3006.t1
High	EISSDGNVNTVDVIFPAHPAYLYTN	10	1	1	g3995.t1
High	cPETLGSHFAPLSGQLK	1	1	1	g3307.t1
High	TYPLEGTMVFFPQYAPYTDPIR	2	1	1	g4355.t1
High	FDGILGLGFDTISVNK	1	1	1	g916.t1
High	AGATAVDSYITSDELIYWYRPTPK	4	1	1	g6554.t1
High	GINVQmGPVVGPIGR	1	1	1	g3139.t1
High	VGAGIVGYQIMEAAHEK	1	1	1	g10153.t1
High	SLNYIPVQDLDYVGFGTmTR	2	1	1	g6145.t1
High	VTLFEDPALSSDSFR	1	1	1	g7147.t1
High	AEASVSNNVPTWR	1	1	1	g10240.t1

High	VAGTAGGETNIDSTRGPYNEGGLY	1	1	1	g7406.t1
High	ALVEGANFATSLGK	2	1	1	g8259.t1
High	HSNYELmLNANDGSK	3	1	1	g9312.t1
High	AIHQLITLVDPLSQSR	1	1	1	g3185.t1
High	YmWADFmLDDPR	1	1	1	g9487.t1
High	LAYSTDLGTTWLK	1	1	1	g8260.t1
High	ATSDSGIFSEmQR	1	1	1	g6784.t1
High	VLIcGGSTPYGGDAIDNcVSIEPDVI	1	1	1	g4355.t1
High	LAVTAGIGSDHVDLDAANK	1	1	1	g8017.t1
High	mSTYPQmTNDAYSPSQTFSHDDIK	2	1	1	g4076.t1
High	GGNKPSFGAGLAGLEPK	1	1	1	g2283.t1
High	NTGKVAGVEVPQLYVSLGGPSDAP	1	1	1	g377.t1
High	DGNVDYDETWGLLNHDWSDWR	1	1	1	g5860.t1
High	GYAPYVQGNPQYPVYR	2	1	1	g8428.t1
High	DKLIWPGDFGISVPAVFLSTNEVDT	1	1	1	g9487.t1
High	SPSSDGDSYNDLPYLPGLLTLSK	2	1	1	g3707.t1
High	DWSDSHYTGPAFK	2	1	1	g10153.t1
High	DSQAHGVFLSNSNGmDIK	1	1	1	g8295.t1
High	NNATGQITLITAR	1	1	1	g10298.t1
High	GIQQSDDDGVVKFETLFPGHYTGR	2	1	1	g2003.t1
High	VALGAFVLPNAK	1	1	1	g7031.t1
High	LLDEATFQTIR	1	1	1	g4355.t1
High	VNTYWYILQDAGK	1	1	1	g10182.t1
High	GDGVTDDAASLNTILR	1	1	1	g8428.t1
High	HYIGYEQETQR	1	1	1	g9150.t1
High	AcDYQAGLTAcPSNFmPLAPPAGLR	1	1	1	g4013.t1
High	AHESLTPGQLSHGTIDIPDGNTNR	3	1	1	g216.t1
High	GYQILLLDQR	1	1	1	g7845.t1
High	RFNGDDSLGDSSR	2	1	1	g5915.t1
High	DTVLSAVTQSR	1	1	1	g6580.t1
High	DLSVWDVVVQDWK	1	1	1	g3139.t1
High	ESSGSIVWESDPNR	2	1	1	g2140.t1
High	FIEIVTWNDYGESHYVGPLDSPHTI	1	1	1	g6554.t1
High	TAAIADNTALTGR	1	1	1	g4461.t1
High	SQYYLSFMSEVLK	1	1	1	g8384.t1
High	FVELmNSmLEDNFLGYVASATSPII	3	1	1	g5915.t1
High	GDGTTDDTAAIQK	1	1	1	g3077.t1
High	SDDPNDFWTSGSSR	1	1	1	g4148.t1
High	TDLADIFFSTK	1	1	1	g727.t1
High	TSSGDPNYGWSTSK	1	1	1	g377.t1
High	QNSSSGSTTTLQVHLENK	1	1	1	g1180.t1
High	TAINNLYNAPAR	1	1	1	g4148.t1
High	SNVPVPAIAIGDYALDLSAFTK	3	1	1	g6147.t1
High	VFAFEVDGYGGR	1	1	1	g6658.t1
High	ALmQLNFETLLK	1	1	1	g10298.t1
High	SLTcVVSSSVSSSK	2	1	1	g7641.t1
High	ITFLSPITPDDFLR	25	1	1	g3995.t1
High	FNAGTESVLAR	3	1	1	g6145.t1
High	DSAGWNQLIVQK	1	1	1	g7045.t1
High	GPcVGNTAAPSGISYPSLcVQDSPL	1	1	1	g3821.t1
High	AAYYSLPEWFNPAYEK	1	1	1	g3954.t1
High	WAmLGAWYPFYR	2	1	1	g8295.t1
High	SPYAYLANPEDER	1	1	1	g216.t1
High	QcIANNPNDLDAQK	2	1	1	g9878.t1
High	HYIGNEQEHFR	3	1	1	g377.t1
High	TTTQFSHENVFTPVFTNEAK	1	1	1	g3149.t1
High	IESDKVDAWFEEDER	1	1	1	g6249.t1
High	ADPSWELPLYQR	3	1	1	g7641.t1
High	NNLRQEGTFAAVEFLNPK	1	1	1	g216.t1

High	ELVSLTLPDIGNTIGSR	1	1	1	g3954.t1
High	NTPIYQDSNTIAmR	5	1	1	g3279.t1
High	IKEQIDGmALSK	5	1	1	g4076.t1
High	SAGSVDLYEGTDR	1	1	1	g4318.t1
High	VDAAAWIQNK	1	1	1	g5993.t1
High	FLLTIAcPAGASNYQK	1	1	1	g466.t1
High	ILTPYYYLNQDSK	1	1	1	g9150.t1
High	LWQYAQSIAASYPDDQR	1	1	1	g4148.t1
High	ILcPETQNTIILIPIENLSPR	1	1	1	g7294.t1
High	VHYDDGQLVYSAGSDPR	1	1	1	g2465.t1
High	YSDNISVYPDGLVLFTYN	3	1	1	g9324.t1
High	VLLYNSDYYTTGTR	1	1	1	g9236.t1
High	LDTLADIPVEDGGIPGSEK	2	1	1	g3728.t1
High	IFSYLDTQLNR	1	1	1	g9148.t1
High	DKFQVPINFNSWTR	1	1	1	g6857.t1
High	SALFLLQSDGK	2	1	1	g7045.t1
High	GIQEAGVIAcAK	1	1	1	g6857.t1
High	DAEEEPYDWSNEGR	1	1	1	g6145.t1
High	LNIFGFPGDPNIR	1	1	1	g10240.t1
High	TAAcVEILQQK	1	1	1	g7294.t1
High	DLVDQLYDSPR	2	1	1	g8384.t1
High	VQTSFGALSVPR	1	1	1	g7406.t1
High	TTIFYSLAGDIR	3	1	1	g5915.t1
High	SDcSDSNLLGSFSENHDNPR	1	1	1	g2159.t1
High	TALVDASGVASLLGTTEVAIVDAPE	1	1	1	g2313.t1
High	DFAFNALNQNFIVPGScPNADQIDA	2	1	1	g5593.t1
High	NLVLDLTAIPASGGSTGIHWPTAQA	2	1	1	g9324.t1
High	VVGESYPTImGSGSK	2	1	1	g9324.t1
High	ATYVGVSTPQTTFHTYTVNWQR	2	1	1	g2989.t1
High	LAALAATVGVDEK	1	1	1	g8672.t1
High	VLTSASVPSDSEVLVEYTG	1	1	1	g1197.t1
High	STSSSSSNYNLLPYQPGLLTLA	1	1	1	g7927.t1
High	LALIQLASGADK	1	1	1	g2727.t1
High	YQYGGYAFYDTK	1	1	1	g9290.t1
High	YVEGPWGGGNAmVPHmSGTSIDA	1	1	1	g8017.t1
High	DTLYSLASGAGK	1	1	1	g727.t1
High	STGAGSLSVWMHNFNDIQYK	1	1	1	g10153.t1
High	LGDFTASPDSR	1	1	1	g8103.t1
High	GYGGDVWFPQPAPK	1	1	1	g8017.t1
High	NALESTEPSVEAK	1	1	1	g2794.t1
High	mSTYPQMTNDAYSPSQTFSHDDIK	2	1	1	g4076.t1
High	GAHVALGPVAGPLGR	2	1	1	g9150.t1
High	SLFQDAINKVNTASNNK	1	1	1	g10182.t1
High	FGVSVGPYSDSFK	2	1	1	g4355.t1
High	TSSADYDADMQR	1	1	1	g6554.t1
High	KIPSELPFLTNSVR	1	1	1	g5593.t1
High	SLcmQDSPLGVR	2	1	1	g377.t1
High	DHGSFIQVDGTSASAPTIAAIISDLN	1	1	1	g8103.t1
High	ASNLDFSQWAGK	1	1	1	g5860.t1
High	GTGLSSTITSQTLAR	1	1	1	g7845.t1
High	VDDLLVFLLDR	2	1	1	g4603.t1
High	GPGFSGSGSQDQGTK	1	1	1	g608.t1
High	TASSLSAALDAK	10	1	1	g3995.t1
High	GNIEGSTPQTPNYYR	1	1	1	g4704.t1
High	FELSLVGNEDVK	2	1	1	g9148.t1
High	YVTTDcGWTVADR	1	1	1	g2125.t1
High	IALEEAFALPR	2	1	1	g4411.t1
High	KGWDAVSGWGTPNFPK	1	1	1	g9511.t1
High	GFPDVSAQAHAYIVR	1	1	1	g8103.t1

High	SYPIYQDSNNLAIR	4	1	1	g2159.t1
High	TGTLEDAVDTR	1	1	1	g456.t1
High	SASPIHLQPVNAAGER	3	1	1	g1116.t1
High	NLFASSmmQATR	1	1	1	g9290.t1
High	TDGQGNAVGSVQDK	1	1	1	g2572.t1
High	IGPAVATGNTVVLK	1	1	1	g5497.t1
High	NNmcDFVNAFDFANPDYSVPSVPT	1	1	1	g9673.t1
High	LGISGYLEQYAR	1	1	1	g8103.t1
High	LVDPLADAATcK	1	1	1	g608.t1
High	GLESALQVSR	1	1	1	g2178.t1
High	NVLAFTILSDGIPIIYAGQEQHYSGS	2	1	1	g2159.t1
High	FHPLPLGSDIPSDDPLAK	1	1	1	g4148.t1
High	HTNTIFASSTK	1	1	1	g6228.t1
High	LSVQQILTPAIELAEK	2	1	1	g2465.t1
High	SINGYPLPGGAWAR	1	1	1	g6145.t1
High	DNIAAFGGDPDR	1	1	1	g10240.t1
High	HALDLDFVHGcAPK	1	1	1	g8480.t1
High	NFVTDETLDLLVK	1	1	1	g5215.t1
High	GAGPNFGAVTSAVVK	2	1	1	g2283.t1
High	NEAYYQKFPEDAER	1	1	1	g7845.t1
High	mIEDNDSEQPFR	1	1	1	g7148.t1
High	QNVPIPSYLFAVASGDLTEAPVGPR	1	1	1	g7147.t1
High	AVAEVYGSSDSQEK	1	1	1	g4318.t1
High	NTPIYQDSNTIAMR	1	1	1	g3279.t1
High	AVTVGASTLADER	1	1	1	g2575.t1
High	SVDPSSLGIDPDVK	2	1	1	g3202.t1
High	TLNDLVAQATK	2	1	1	g1372.t1
High	NVSNEPmQVDGK	1	1	1	g5215.t1
High	SLGAAEDVNLVR	1	1	1	g7301.t1
High	AAASKPAALVDSSGK	2	1	1	g3077.t1
High	LNFVTGSNVGSR	1	1	1	g2234.t1
High	LIDNFVIDLENSLGVK	1	1	1	g7294.t1
High	AAEIPSFVWLDTAAK	1	1	1	g5707.t1
High	DLLVGPPLTISAVQVLK	2	1	1	g5328.t1
High	YYGASIPTEDFSTESLR	1	1	1	g5245.t1
High	TAVWSEIPQLGK	2	1	1	g2384.t1
High	IAVQNSIVLPTQYSWTHR	1	1	1	g3685.t1
High	LNLLLDAAGEYK	1	1	1	g7148.t1
High	AWSDALEWSGHEK	2	1	1	g3202.t1
High	TPIVEYAEQDGR	1	1	1	g1325.t1
High	TIYNVGDAFLPK	1	1	1	g9290.t1
High	AVGSDYWDVDETGR	1	1	1	g10153.t1
High	SLcMQDSPLGVR	1	1	1	g377.t1
High	GVmTILDNHVSR	4	1	1	g5860.t1
High	NWVFPSDLLWYNR	1	1	1	g6554.t1
High	TNIAEWDYGTK	2	1	1	g3995.t1
High	DGSTGPFSVIALR	3	1	1	g1116.t1
High	YAATELEDLEIVDNKHK	1	1	1	g3202.t1
High	NANLYSSHPIYLEmR	1	1	1	g9290.t1
High	NEISDIAEPR	1	1	1	g1180.t1
High	YYcAVFLIDPK	1	1	1	g1079.t1
High	SAFAIVNTGAIR	1	1	1	g8480.t1
High	FAYYTSDYSEAK	1	1	1	g3279.t1
High	LGGLmYcTPTER	1	1	1	g4461.t1
High	TAEFASSWYNR	1	1	1	g3954.t1
High	LYHSEAILLPDGR	1	1	1	g4355.t1
High	GPYFLENDAGTK	1	1	1	g216.t1
High	TSSADYDADmQR	2	1	1	g6554.t1
High	LRTSLPSNPNIYGLGEDSDSFK	1	1	1	g8295.t1

High	VQSDFESEFK	1	1	1	g10182.t1
High	AGmASImcSYNR	2	1	1	g9150.t1
High	AFNHYFGTmAGVR	1	1	1	g9673.t1
High	TATQEFQIAVLGPEEPQRPQPVR	2	1	1	g4704.t1
High	SQSYVYITPTYLR	2	1	1	g8384.t1
High	NHADITAPFQEFYR	1	1	1	g9290.t1
High	IANVLNETDDAEK	1	1	1	g5548.t1
High	TSILAPGDSETLSLPVTR	1	1	1	g3139.t1
High	LSYENIPTVVFSHPEVGTIGLTEPQA	1	1	1	g3006.t1
High	VLmVLYDGGEHAK	1	1	1	g8017.t1
High	YVESGLNDHGDR	2	1	1	g1180.t1
High	ALGVTQLFAPLADLAR	1	1	1	g6753.t1
High	SNFHPVGTASmLPR	1	1	1	g2143.t1
High	YLQLTPTDAVQGR	1	1	1	g3578.t1
High	GQDNTVQFVR	1	1	1	g6228.t1
High	LGNTDFYGASK	1	1	1	g2234.t1
High	VDmTTAQHTSLFR	1	1	1	g6145.t1
High	AGVDTDFSEIR	1	1	1	g6537.t1
High	ALMQLNFETLLK	1	1	1	g10298.t1
High	IDVSSWDVVK	1	1	1	g9150.t1
High	TAYYPTSPSSTISPLGANcAIPLGAP	3	1	1	g7045.t1
High	ELYDVDEVLFTTDINPTLAHLASK	1	1	1	g7148.t1
High	FASYTSDFSLAK	1	1	1	g2159.t1
High	SILDTLPGAKPLSK	1	1	1	g8384.t1
High	STYAQNVAAmLQSTGLDGVDIDWI	1	1	1	g3385.t1
High	SLVDTQQHLGWLPDcR	1	1	1	g6145.t1
High	NVDGALPLTGSER	3	1	1	g377.t1
High	IKPPQIGSYGQILEWR	1	1	1	g4461.t1
High	AADSDGEEYTAR	1	1	1	g4965.t1
High	WEELLTLGPR	1	1	1	g6554.t1
High	ILSQTAVDLIFTNFNAR	2	1	1	g4704.t1
High	DIQAWEYVPLGPFNAK	2	1	1	g6147.t1
High	FIEAQLDAYEK	1	1	1	g7206.t1
High	LASASSQGYPALR	2	1	1	g4461.t1
High	IYVTGESYAGR	1	1	1	g699.t1
High	GGNIVIPDQSWHTWR	2	1	1	g1839.t1
High	IGISFISIDTAK	1	1	1	g9337.t1
High	NVQYVQTFTDR	1	1	1	g9063.t1
High	AYIDSIVAQLK	1	1	1	g5707.t1
High	SQSIYFLLTDR	1	1	1	g3279.t1
High	DEVDTYVVSLVQSLGTEGK	1	1	1	g5705.t1
High	QLAANVFPNPVTSR	1	1	1	g7206.t1
High	GKLPAGSVITVEPGVYFcR	1	1	1	g7148.t1
High	ATVNLGFIDVAR	1	1	1	g5625.t1
High	AHFALWAImK	2	1	1	g2384.t1
High	HIDGFGVHTYR	1	1	1	g9148.t1
High	SDSTLQIFQIR	1	1	1	g7627.t1
High	ASSIVVSGTPIIRPTGQILANPTATP	1	1	1	g6147.t1
High	AGFAGDDAPR	1	1	1	g8069.t1
High	GGVNDLLVPFLK	1	1	1	g4734.t1
High	QNELLVSEFADR	1	1	1	g456.t1
High	LDETVWcIR	1	1	1	g6836.t1
High	DNAcTATNPPAPAAGSGTHIK	1	1	1	g9076.t1
High	IAWVGEQSAIPNK	1	1	1	g3307.t1
High	LQSSQSGQLNVK	1	1	1	g4461.t1
High	YTLEQIIDAVR	1	1	1	g5733.t1
High	SWEVVYDAYK	2	1	1	g2283.t1
High	AGQTATNKPVLmFALPHHVESFDA	1	1	1	g10078.t1
High	SIYFLLTDR	1	1	1	g2159.t1

High	ADPDYYYTWTR	2	1	1	g2158.t1
High	FNGDDSLGDSSR	4	1	1	g5915.t1
High	SGIYTIQAPVR	1	1	1	g2794.t1
High	NDLVFGSNAELR	1	1	1	g4318.t1
High	NPYFSTWLANAR	1	1	1	g456.t1
High	DQPNEFGEYPGYR	1	1	1	g6228.t1
High	ALVVSVDEHPmWIYEVDGR	1	1	1	g7487.t1
High	NQDVYmPLSGLR	1	1	1	g7301.t1
High	YIDQNETEPVR	1	1	1	g6228.t1
High	DAIIQADQNR	2	1	1	g571.t1
High	NSPTGIAASAGIGK	1	1	1	g10182.t1
High	VTGILFENK	1	1	1	g10078.t1
High	TASGDISYDPAAGGIHSAQLAR	1	1	1	g8260.t1
High	LTGDLVFWK	1	1	1	g7783.t1
High	VLDPLVAGPAR	2	1	1	g2149.t1
High	FTDLGLNTVR	2	1	1	g608.t1
High	LHIAEIPTISLS	2	1	1	g5915.t1
High	TSALSFLSYSTK	1	1	1	g395.t1
High	TSNVGTFGTPLKK	1	1	1	g9148.t1
High	YLGSGQDWYR	2	1	1	g4474.t1
High	cINWAGHIDLR	1	1	1	g5860.t1
High	VLVSGSDPEDATHPEEYR	1	1	1	g4355.t1
High	DLDFLNTNYK	5	1	1	g3995.t1
High	TILAAYYNEAR	1	1	1	g4355.t1
High	EGGPVFLLESGETSGEDRLPYLEK	1	1	1	g5245.t1
High	AQImDYEATR	2	1	1	g1180.t1
High	GVAAYLFER	1	1	1	g216.t1
High	SPYAYLANPEDERAQYEYDTDKTm	1	1	1	g216.t1
High	YLGEDVFLQGVR	1	1	1	g7301.t1
High	QTYVDAATTLR	1	1	1	g4148.t1
High	VVGESYPTIMGSGSK	2	1	1	g9324.t1
High	TmHELYLWPFADAVR	1	1	1	g6857.t1
High	TNIAEWDYGTKGDLSYHK	2	1	1	g3995.t1
High	LILSSDSKTEVVQSR	1	1	1	g1180.t1
High	TFYGTYDYVPQAIDR	1	1	1	g9511.t1
High	IKEQIDGMALSK	1	1	1	g4076.t1
High	ASGSLYLDDGK	2	1	1	g8295.t1
High	YSYMIHAEDADWQTK	1	1	1	g4633.t1
High	GRTTIFYSLAGDIR	1	1	1	g5915.t1
High	WNYLEYPYTR	1	1	1	g6228.t1
High	ALNTNYDGSAHGVFTK	1	1	1	g4318.t1
High	TWYLAYAAGLK	1	1	1	g10078.t1
High	GSTLLEDFIFR	2	1	1	g9148.t1
High	IDFSLPVPmVER	2	1	1	g3149.t1
High	GQQQVEcTAK	1	1	1	g1079.t1
High	DILPITPGVVEGWGK	2	1	1	g4965.t1
High	ELVDYLHENDQHYVVmVDPPISVD	1	1	1	g9290.t1
High	IISNTQANK	1	1	1	g2143.t1
High	GPLNEGGLYAER	2	2	1	g4603.t1
High	SITVDYLSLPcK	1	1	1	g5245.t1
High	FTAIFGSDAR	1	1	1	g6857.t1
High	LNILHWHLDDSQSWPVR	2	1	1	g4076.t1
High	YRVEDVWTGK	1	1	1	g2384.t1
High	cGLPGDFDTPEYAR	1	2	1	g3954.t1
High	ILPFTVPGSPINEQGLK	2	1	1	g8384.t1
High	TIDESIVVVQQPEESWK	1	1	1	g8261.t1
High	DNTELVYLR	1	1	1	g8260.t1
High	NAIISDAWER	1	1	1	g4076.t1
High	LEPVDHQIILNTGHTTINGVSR	1	1	1	g7482.t1

High	SIYNDALWNR	1	1	1	g1180.t1
High	NTPIYQDSNTIAmRK	2	1	1	g3279.t1
High	GIWYDFWTGEK	1	1	1	g9290.t1
High	SNYWQPLLYHIR	1	1	1	g4013.t1
High	VAELFFDVPLNYSKPNEER	1	1	1	g7845.t1
High	VLDGSGNVVK	1	1	1	g9990.t1
High	YNGENYcSLWR	1	1	1	g571.t1
High	NFGAFNDVWVR	1	1	1	g2927.t1
High	DLEDAVSGYK	1	1	1	g3728.t1
High	NPFSFAIQR	3	1	1	g8295.t1
High	NLLVATIQER	1	1	1	g2149.t1
High	VVDASSFPFLPPGHPQALVYALAEK	1	1	1	g3369.t1
High	SEEYEVVHR	1	1	1	g8480.t1
High	VTDTIYIPPGSR	8	1	1	g9324.t1
High	EGYENLTTAPK	3	1	1	g7148.t1
High	ISLLGYVER	1	1	1	g3669.t1
High	IVLGVLTAATAGNSWQPTTVLSTTI	1	1	1	g9063.t1
High	FSTPGVSGALVPR	3	1	1	g727.t1
High	ENLAVASTDNAK	1	1	1	g10182.t1
High	DISSGDLIR	1	1	1	g3707.t1
High	VPTWATGSSFFSINSGSHEDVLADS	2	1	1	g5915.t1
High	FNYWFPLDGDKK	1	1	1	g2283.t1
High	NTDSVLPLSGK	1	1	1	g6857.t1
High	DELGTLGQAR	1	1	1	g4734.t1
High	FDQDGVTIR	1	1	1	g2149.t1
High	ILPVEDVPITPLGK	2	1	1	g9148.t1
High	NGATVFQGTSLK	1	1	1	g6554.t1
High	ANVAGAGTSVR	1	1	1	g4318.t1
High	FVFNTEAHPFK	3	1	1	g4633.t1
High	DHSDNTSPPHELYR	1	1	1	g1266.t1
High	TLHELYLWPFYEGVK	1	1	1	g9150.t1
High	FSATTELYR	3	1	1	g5993.t1
High	DIYKPYIQAYK	2	1	1	g9061.t1
High	QGIWPAFWILGDSLR	1	1	1	g1839.t1
High	LNDNFGVPK	1	1	1	g8384.t1
High	NLPLQQTLALK	6	1	1	g377.t1
High	GNAFFQGDTR	1	1	1	g608.t1
High	VGVSLVSTEK	1	1	1	g4882.t1
High	LLYETGLPVR	1	1	1	g6249.t1
High	TGQNVYDVR	1	1	1	g3202.t1
High	SQYYLSFmSEVLK	1	1	1	g8384.t1
High	FGTNAIANK	1	1	1	g1839.t1
High	AVSFSSGGFSER	1	1	1	g8103.t1
High	DVAIQYSETVGK	1	1	1	g1266.t1
High	LLVEYQTNSR	1	1	1	g8295.t1
High	IPVPELWIDILEK	2	1	1	g7406.t1
High	STDTPVFFIR	1	1	1	g1180.t1
High	WPLVTAAAQK	1	1	1	g8295.t1
High	TFGESLTLLR	1	1	1	g290.t1
High	TSAYWQNLFK	1	1	1	g6145.t1
High	GIQQSDDDGVVK	1	1	1	g2003.t1
High	RPAERPFIISR	3	1	1	g8295.t1
High	LTFWTSLDAK	1	1	1	g8260.t1
High	QILGLISTAASK	1	1	1	g2149.t1
High	GSWLVNTAR	1	1	1	g8017.t1
High	NILDSYFSGR	1	1	1	g8017.t1
High	TTAGLNAWHGGDDLVVK	1	1	1	g3139.t1
High	EATPFADDAPGPR	1	1	1	g5245.t1
High	NGVTYTVFEHAATGAK	1	1	1	g4362.t1

High	VTIVNSPFSFK	1	1	1	g1266.t1
High	NDSGTFFIK	1	1	1	g8248.t1
High	DNGIFVNSR	1	1	1	g6145.t1
High	LGAGmLTAER	1	1	1	g7845.t1
High	EYTVQLEAHDVAVLK	2	1	1	g2384.t1
High	HQGYLPDGR	1	1	1	g9337.t1
High	DALVSVQDQR	2	1	1	g2384.t1
High	DFVDTVTGDR	1	1	1	g5495.t1
High	QEYDESGPSIVHR	1	1	1	g8069.t1
High	EISSDGNTQTIDVIYPAFPFFLYTSP	1	1	1	g456.t1
High	YSGGQEGVDAK	1	1	1	g4318.t1
High	VLQLSSGDcSK	1	1	1	g4461.t1
High	IALPSFDEEAVLK	2	1	1	g4878.t1
High	DLQLmNIAR	1	1	1	g7294.t1
High	STFISQINR	1	1	1	g9148.t1
High	FFGEFPNLR	1	1	1	g10240.t1
High	TLPNIPGAVNNDLGGR	1	1	1	g4355.t1
High	APNPGEILK	1	1	1	g2465.t1
High	SPAGANQWVAK	1	1	1	g4318.t1
High	QWQFDISDALK	1	1	1	g3149.t1
High	LFTTVAALR	1	1	1	g4704.t1
High	YGLPLDSR	1	1	1	g456.t1
High	VPSWIVSPWTR	1	1	1	g9673.t1
High	YFALSQFTR	1	1	1	g727.t1
High	ILDFADISWSTNR	1	1	1	g6921.t1
High	GVDmGAEFR	1	1	1	g9150.t1
High	TIETWYGR	2	1	1	g10375.t1
High	YDPDYFFYALK	1	1	1	g10153.t1
High	LISLVKPFTDALTELGIK	1	1	1	g3187.t1
High	YDDLDTFLK	1	1	1	g8103.t1
High	YWLQAQDHTGEAR	1	1	1	g8428.t1
High	GNmmLALLR	1	1	1	g10078.t1
High	LIGAESVLGR	1	1	1	g2572.t1
High	NAGKPcLLEEYGSK	1	1	1	g1408.t1
High	VmLmmGGAAR	1	1	1	g9063.t1
High	GDDLWADR	1	1	1	g4745.t1
High	EVILSAGSLK	1	1	1	g2143.t1
High	LFGPPEFK	1	1	1	g3006.t1
High	VGAGIVGYQImEAAHEK	1	1	1	g10153.t1
High	DAVTYTEHAK	1	2	1	g2935.t1
High	QVIGSLTEGR	1	1	1	g9673.t1
High	DFLPYYIATFK	1	1	1	g4745.t1
High	VAGSTTGLR	1	1	1	g9324.t1
High	ELPGFPDDLQPPSAK	1	1	1	g9290.t1
High	YVSNSYVR	1	1	1	g5915.t1
High	SPLVIGAALK	1	1	1	g2125.t1
High	DGmTPVTVK	2	1	1	g608.t1
High	DcAHGSDVSTFVDAVEK	1	1	1	g6836.t1
High	YSDSSQLR	1	1	1	g7540.t1
High	NPTGTIHEK	1	1	1	g4411.t1
High	IVHAANLDGR	1	1	1	g7045.t1
High	TTWGVFLTVR	2	1	1	g10298.t1
High	IDVGYVAHPSFVEADELK	1	1	1	g10174.t1
High	QSNWYHFVR	1	1	1	g456.t1
High	ASLLIPGR	1	1	1	g3307.t1
High	VAEDGVTPLGDAVQLIDRDDSDGP	1	1	1	g4208.t1
High	NVDNFSFLK	1	1	1	g4362.t1
High	SLDSYEFK	1	1	1	g7147.t1
High	SPDPNTGLSR	1	1	1	g4148.t1

High	ASSTLPPTPDEDLcNcmSK	4	1	1	g7641.t1
High	RTLmLDSGIYR	1	1	1	g4965.t1
High	FFVYDQSR	1	1	1	g4633.t1
High	FSDSDDHVLQQFK	1	1	1	g7301.t1
High	IDSLAAIGK	1	1	1	g4965.t1
High	QLEFLLGR	1	1	1	g8294.t1
High	SGVTTNIQK	1	1	1	g4603.t1
High	FNWGADEVK	1	1	1	g6228.t1
High	LPEGVTAPDILPGLLKR	1	1	1	g290.t1
High	SLFQDAINK	1	1	1	g10182.t1
High	SGHWTGDNISSWDHYR	1	1	1	g9290.t1
High	TVPVTQSFVWSGNK	1	1	1	g6228.t1
High	QDDLTQDWR	1	1	1	g5245.t1
High	VYGVQNLR	1	1	1	g3369.t1
High	TLHEIYLWPFAEGVK	1	1	1	g3139.t1
High	DSAFLTYK	2	1	1	g3279.t1
High	SSGNLTFmR	1	1	1	g3202.t1
High	TLTAAEAGDYFPQTPmRLR	1	1	1	g2989.t1
High	LTIFSGEVHPFR	1	1	1	g4603.t1
High	FSLVSGGVR	1	1	1	g3578.t1
High	TLmLDSGIYR	1	1	1	g4965.t1
High	QTLLSSIHGSSLDVR	1	1	1	g8260.t1
High	VDAWTGGARPR	1	1	1	g9487.t1
High	APEVEDLQLHIPGVK	1	1	1	g3006.t1
High	TVYVGNSSR	2	1	1	g377.t1
High	GAIVVKEDVAEAVK	1	1	1	g8017.t1
High	AYSPPAYPAPWASGAGEWAQAHD	1	1	1	g377.t1
High	HLQLAIR	1	2	1	g1937.t1
High	TSIVDTAVSR	1	1	1	g6753.t1
High	LGDWPNFPDFTVPGV	1	1	1	g4355.t1
High	AWVNmLVR	2	1	1	g10078.t1
High	APIGDRPSLAEIEEALK	1	1	1	g290.t1
High	SSSVAIVAR	1	1	1	g7811.t1
High	SAGTELSAR	1	1	1	g2140.t1
High	YWEVGGVR	1	1	1	g7148.t1
High	GNLGIAFYAK	1	1	1	g3385.t1
High	GTLVGPNVYSSIGVLGITGGHSDIH	1	1	1	g3307.t1
High	YIDADILEK	1	1	1	g7148.t1
High	AASIYLDALK	1	1	1	g1180.t1
High	GIYWcPWcDGYEHR	1	1	1	g4965.t1
High	GVLLDVSR	1	1	1	g2794.t1
High	FVTNDGSSK	1	1	1	g9148.t1
High	NLVPAASEDTR	1	1	1	g4226.t1
High	QQHIDLLSSGNHELYK	1	1	1	g8480.t1
High	VSLDSNGKPTGSR	1	1	1	g3685.t1
High	FATLVYTVHELANNPDLAASTLDGL	1	1	1	g10078.t1
High	VLIDLFR	2	1	1	g2140.t1
High	AQFQGLSGNNHFANQNEQHGSLK	1	1	1	g10326.t1
High	ELETPGAGSAHDSSTAGLIK	1	1	1	g5215.t1
High	EIGFTVQEGSLTEHVVK	1	1	1	g3149.t1
High	TWTLDPERFPLHK	8	2	2	g8295.t1;g9290.t1
High	IVLGSQQLALTKPFTLAEHEEK	1	1	1	g1180.t1
High	RIVLGTGmKDILPITPGVVEGWGK	2	1	1	g4965.t1
High	LFLLGYNDLSVGSLSNWADR	1	1	1	g5446.t1
High	NWEGFAPDPVLTGQMMASTIEGM	1	1	1	g377.t1
High	KQPDYANYPAER	1	1	1	g8428.t1
High	SRVTFDSQISTTQVR	2	1	1	g5548.t1
High	LPEHEPAGDWLVTYDVGTR	1	1	1	g10375.t1
High	DGHNYATLNTALVAPLSR	1	1	1	g3369.t1

High	EDIDALAVPVQFLAPEIDPVYTPELK	1	1	1	g9160.t1
High	YVNIDDcWSVK	1	1	1	g2384.t1
High	SYANSQLSGLTK	1	1	1	g1751.t1
High	WQLYPTLAGYDSK	1	1	1	g5712.t1
High	AIKDAYEVAmLR	2	1	1	g7148.t1
High	SGGTcVNVGcVPK	1	1	1	g3006.t1
High	IVTGPLYLPR	1	1	1	g7031.t1
High	QYTLTIPQGYNPSEPYK	1	1	1	g9076.t1
High	GSEEELLEKR	1	1	1	g7148.t1
High	SLHDEPDNVAmIK	1	1	1	g8248.t1
High	NVmDDANIPSLLALPYLGFLDVDDE	1	1	1	g6658.t1
High	DSYVGDEAQSK	1	1	1	g8069.t1
High	ALYLGPEVPAEVLTWQDPVPAVNH	1	1	1	g4318.t1
High	NLVATADDVDGTK	1	1	1	g4567.t1
High	EINQHVDVR	2	1	1	g377.t1
High	WAMLGAWYPFYR	1	1	1	g8295.t1
High	SAPDAPQVLFPGDAR	1	1	1	g5733.t1
High	LLESEAYFR	1	1	1	g9290.t1
High	GSNFIPPDAFWPR	2	1	1	g3149.t1
High	RImGAVHGEAHR	1	1	1	g7147.t1
High	FNADmTAYTGAWGRPQR	1	1	1	g8259.t1
High	VQPKPFTVSIPDEQIEELK	1	1	1	g7783.t1
High	QALPFNGSDTPK	1	1	1	g7294.t1
High	INEELLAK	1	1	1	g3993.t1
High	IGEDNLPQR	3	1	1	g4474.t1
High	ALVEGSTFAGR	2	1	1	g2140.t1
High	SGKDANSLLGTIHTFDPEAAcDDTT	1	1	1	g8259.t1
High	mVLGmPLYGR	3	1	1	g466.t1
High	SVAIVEmQDR	2	1	1	g10298.t1
High	LKSQVLDSVNYVLSHQQADGWLGI	2	1	1	g5915.t1
High	SGGTIGPmTSAQIGLR	1	1	1	g7627.t1
High	TSHcPYKGEASYYVVK	1	1	1	g6249.t1
High	KGFFWAQK	1	1	1	g9487.t1
High	ELLLEAGxPDLF	2	1	1	g6857.t1
High	VSAGEWVNR	1	1	1	g6537.t1
High	QEYVEQQPGmR	1	1	1	g4461.t1
High	HLQEETTVDTVYDmASLTK	1	1	1	g4704.t1
High	RDTLYSLASGAGK	1	1	1	g727.t1
High	SREDAEALVSR	1	1	1	g2149.t1
High	GGVDYVNK	1	1	1	g881.t1
High	SLGLDVAFPPL	2	1	1	g4704.t1
High	LSYQVVPVTR	1	1	1	g571.t1
High	EIGSASIVLLK	1	1	1	g377.t1
High	QAAQNIWK	1	1	1	g3185.t1
High	QFSGPADLAGK	1	1	1	g1325.t1
High	VDLVFWDK	1	1	1	g1325.t1
High	NPDDIVNDIK	1	1	1	g10182.t1
High	AEDLPPSmDSYAF	1	1	1	g3185.t1
High	DQmQYWYR	1	1	1	g4745.t1
High	HAFEITDLTDQR	1	1	1	g4411.t1
High	AVLTTcESTVPTVNG	1	1	1	g2392.t1
High	EINQHVDVRGDHAK	1	1	1	g377.t1
High	LVDKDFQEGSYGGHK	1	1	1	g1180.t1
High	TWSSNNIVR	1	1	1	g4704.t1
High	RIDVSSWDVVK	1	1	1	g9150.t1
High	QLTEDLATFK	1	1	1	g9148.t1
High	EIGGASAVLLK	1	1	1	g9150.t1
High	LDKmFEPGVAK	1	1	1	g9337.t1
High	KGGSEQTVIR	1	1	1	g8260.t1

High	EDAGIVLEDNR	1	1	1	g9556.t1
High	AINSGVAAAQ	1	1	1	g3385.t1
High	IFAINScDGTLTEcDAYSTTPGDGPI	1	1	1	g7811.t1
High	GVDYQPGGSSK	1	1	1	g608.t1
High	WIGGEWTQ	1	1	1	g3202.t1
High	FHDSSNDPDKR	1	1	1	g8428.t1
High	EDAANNYAR	1	1	1	g4292.t1
High	NVKDFGAKGDGSTDDTDAINSAIS	1	1	1	g3077.t1
High	AQIMDYEATR	1	1	1	g1180.t1
High	GDTFDISR	1	1	1	g4745.t1
High	DLAFDYNSEK	1	1	1	g7206.t1
High	LDNGSPLR	1	1	1	g9063.t1
High	LIDAAFER	1	1	1	g3728.t1
High	LYDEAQLAK	2	1	1	g7845.t1
High	ISLNENSIWSGPFLNR	1	1	1	g4461.t1
High	NPQPPLLK	1	1	1	g3993.t1
High	WPQVADAAR	3	1	1	g9290.t1
High	NIDNALPLSK	2	1	1	g9150.t1
High	ASELLQR	1	1	1	g7540.t1
High	QHGHVPGTHHTELEDNEmIAIDm	1	1	1	g4999.t1
High	IGAASVVLLK	1	1	1	g6857.t1
High	GSAANmSLGGGK	1	1	1	g2575.t1
High	DAGLIAGLNVLR	2	2	1	g2092.t1
High	HAGYLPDcR	2	1	1	g3185.t1
High	QVIVVNK	1	1	1	g6228.t1
High	IPIGTSPTLK	1	1	1	g3993.t1
High	STDYAVLLLTDVIGHK	1	1	1	g10174.t1
High	IDDmAVR	2	2	2	g377.t1;g6857.t1
High	VGAPNGQER	1	1	1	g7487.t1
High	GGGGNAYGVVTK	1	1	1	g559.t1
High	DIPYLTAIR	2	1	1	g7641.t1
High	GAYAVIDPHNYGR	1	1	1	g4348.t1
High	VVLGTDWTAGENGK	1	1	1	g3307.t1
High	LDPLLSPGK	2	1	1	g2392.t1
High	GASLTVEIK	1	1	1	g3149.t1
High	SNFASDPK	1	1	1	g8535.t1
High	SGDLVPLGPR	1	1	1	g8480.t1
High	VLYSVDYPFSANEK	1	1	1	g3952.t1
High	YVDSLPEIK	1	1	1	g4603.t1
High	NGGPNFEQIPINRPR	1	1	1	g9148.t1
High	AGTTSVIAR	1	1	1	g3185.t1
High	FASTGDNPR	1	1	1	g4362.t1
High	SFLENEDSAR	1	1	1	g6228.t1
High	SIFPcQDTPDVK	1	1	1	g7147.t1
High	GGSIIPLR	10	2	2	g9290.t1;g8295.t1
High	GVAmAEEHR	2	1	1	g377.t1
High	ITANISPR	3	1	1	g4950.t1
High	SVFNTPWIYR	1	1	1	g1180.t1
High	FSTVAGSR	1	1	1	g9148.t1
High	TLDRAEFDEGTR	1	1	1	g9490.t1
High	YAVLADVYTEDKTR	1	1	1	g6377.t1
High	ENPLFGWR	1	1	1	g7294.t1
High	TVNDQISR	1	1	1	g7668.t1
High	NPIPmLQIGESGDTGNVEIQDLILE	1	1	1	g3077.t1
High	GFFWAQK	1	1	1	g9487.t1
High	QPYISPPVR	1	1	1	g7294.t1
High	SILDPPSGQQDSSSQQSDLVFEYTK	1	1	1	g8295.t1
High	QSLFYEIDIK	1	1	1	g1079.t1
High	GHFVANGNEK	1	1	1	g4603.t1

High	MAELNESSQLGSLSHQNNNNSPGT	1	1	1	g8921.t1
High	SSGYTDWVNK	1	1	1	g5121.t1
High	NGEVWTK	1	1	1	g3185.t1
High	LSGLSPVR	2	1	1	g5915.t1
High	TAFTGSWGRPQR	2	1	1	g2158.t1
High	LSEALHAR	1	1	1	g3279.t1
High	IDDMAVR	1	2	2	g377.t1;g6857.t1
High	FIEAIER	1	1	1	g7147.t1
High	FASALSNYK	1	1	1	g3373.t1
High	HLLVASSR	1	1	1	g4461.t1
High	FIIEPYLK	1	1	1	g7148.t1
High	DTLFIPPGSR	1	1	1	g8428.t1
High	TWmPTDPYIYGLGEDVDSFRR	1	1	1	g9290.t1
High	VYDLLVIK	1	1	1	g8672.t1
High	NVcHGSDSVENAKK	1	1	1	g7496.t1
High	YFVEDSVGR	1	1	1	g395.t1
High	TAVYPLQLR	1	1	1	g10078.t1
High	NYFLmEDDNLNHPSNFIGNK	1	1	1	g10078.t1
High	SSFYVVR	1	1	1	g4603.t1
High	LLESGHPc	1	1	1	g2159.t1
High	DLPLSGSLK	1	1	1	g9150.t1
High	SLIDTYR	1	1	1	g3185.t1
High	NFITTTK	2	1	1	g4076.t1
High	TPFTWGK	2	1	1	g377.t1
High	FIIEPYLKDSELSK	1	1	1	g7148.t1
High	ScFEIGR	1	1	1	g216.t1
High	IVLGTGmK	1	1	1	g4965.t1
High	IVSLSLTEK	1	1	1	g881.t1
High	ImSAFYK	1	1	1	g377.t1
High	FNVDTTAFTGSWGRPQR	1	1	1	g2140.t1
High	LQGSAVGAGK	1	1	1	g5712.t1
High	DLGcVHK	1	1	1	g2384.t1
High	LFDSIIK	1	1	1	g3149.t1
High	TLIQDLR	2	1	1	g6658.t1
High	SmDNALGR	1	1	1	g3202.t1
High	HESGTSAILR	1	1	1	g456.t1
High	TFVYEcK	1	1	1	g6658.t1
High	AAWAPTVAYK	1	1	1	g6921.t1
High	NLPLQQTLALKIGH	1	1	1	g377.t1
High	DTImVQNNGHAVIR	1	1	1	g7482.t1
High	YcIDQGR	1	1	1	g4314.t1
High	EIPDFDFDSTK	1	1	1	g3185.t1
High	FTPEVLLSAPR	1	1	1	g8539.t1
High	VTFNSEK	1	1	1	g2283.t1
High	DVHGFATR	1	1	1	g9148.t1
High	GLIDIYK	1	1	1	g9337.t1
High	DINIPFSR	1	1	1	g7209.t1
High	ELDDmIK	1	1	1	g8657.t1
High	ATVPSASIESR	1	1	1	g3211.t1
High	IMSAFYK	1	1	1	g377.t1
High	SGATTPTVDTEEIVYWYRPTPK	2	1	1	g9061.t1
High	VYTDSFR	1	1	1	g2140.t1
High	NIYFTVR	1	1	1	g6228.t1
High	LSPYSSYFHTGGDEVK	1	1	1	g2794.t1
High	IFNTFYTSVKPLIAEK	1	1	1	g8672.t1
High	EVGGIAGEIWPAIKK	2	1	1	g3307.t1
High	IIYPTNYISLITNEHQK	1	1	1	g7294.t1
High	ImPYSGGTSR	1	1	1	g6228.t1
High	NAAGTGTVIETSSATGTPAAGTSAT	1	1	1	g9012.t1

High	EIPDFDFESTHSAAVSAWTDK	1	1	1	g6145.t1
High	SVISESGGGR	1	1	1	g1569.t1
High	FSGLTPmGTSLK	1	1	1	g2149.t1
High	AADmILGLQWLVEHDPR	1	1	1	g5915.t1
High	GVmIDTGR	1	1	1	g4076.t1
High	TSGLLSLLK	1	1	1	g7301.t1
High	cSGYELK	1	1	1	g9290.t1
High	HYPYSR	1	1	1	g571.t1
High	SSIPLLDLK	1	1	1	g5993.t1
High	YQVMMQQLQK	1	1	1	g5754.t1
High	SAFRPSDDATIFGYFVPANAMmSV	2	1	1	g6658.t1
High	KESSGSIVWESDPNR	1	1	1	g2140.t1
High	FKAGTTSVIAR	1	1	1	g3185.t1
High	GALVNDTQR	1	1	1	g4411.t1
High	SIPFSAAGQTTFLK	1	1	1	g9163.t1
High	TAVNPKGLPQLTSR	1	1	1	g881.t1
High	TVSLNPNVR	1	1	1	g9455.t1
High	NPWSLQLQcmNDLPQMPK	1	1	1	g4732.t1
High	AQWELLPR	1	1	1	g7147.t1
High	EQIDGmALSK	1	1	1	g4076.t1
High	YSFEEVSK	1	1	1	g6643.t1
High	GSSQTPTVSK	1	1	1	g5328.t1
High	DGLLSLPR	1	1	1	g8117.t1
High	mIAGDATLR	2	1	1	g2392.t1
High	DIPLSSVAK	1	1	0	
High	NDEATASGVVGRPTHGR	1	1	1	g3626.t1
High	ISLPDFSK	1	1	1	g4603.t1
High	SSHPIEVPVKR	1	1	1	g7301.t1
High	SDmVAEQLK	1	1	1	g3187.t1
High	ATAATIDGPAR	1	1	1	g3970.t1
High	ASPEDITNLSK	1	1	1	g6643.t1
High	VLSIATPGLQR	1	1	1	g3385.t1
High	GWDDVTATALR	1	1	1	g4126.t1
High	NGEVVPSQQLK	1	1	1	g3954.t1
High	EAVGYWVGK	1	1	1	g4704.t1
High	TATTSDEKNTALR	1	1	1	g7301.t1
High	AQYEYDTDKTmTmLR	1	1	1	g216.t1
High	HLTGEFEK	1	1	1	g3250.t1
High	WSTEAIR	1	1	1	g4550.t1
High	GLGFGAAEDNVNEYSVPPGc	1	1	1	g571.t1
High	TLASVTKR	1	1	1	g3149.t1
High	SLLGDVAVSPAGDI	1	1	1	g5324.t1
High	TAVITGGGSGLGR	1	1	1	g9459.t1
High	FIHDWTPGK	1	1	1	g1180.t1
High	SAAGNFIK	1	1	1	g10240.t1
High	DAGVDPTGAPR	1	1	1	g5613.t1
High	VTLELGGK	1	2	1	g5497.t1
High	GLNTVFISYAGLTAImFVASLcLYD	1	1	1	g1073.t1
High	DGQAHGVYIASSNGmDIFISK	1	1	1	g9290.t1
High	TNEWETVR	1	1	1	g216.t1
High	VPEQPYHK	1	2	1	g2392.t1
High	NIVYPGLEDK	1	1	1	g5245.t1
High	ETDPEEYPR	1	1	1	g3149.t1
High	GSIPVGLVAGPDGNAWVGLLGNST	1	1	1	g3685.t1
High	LGTIIVGDPLNRPIIK	1	1	1	g8428.t1
High	GmLSFPR	1	1	1	g8260.t1
High	DLEDAVSGYKR	1	1	1	g3728.t1
High	RGVAmAEEHRGK	1	1	1	g377.t1
High	STFAGDGTR	1	1	1	g8295.t1

High	FVHQNEVDGK	1	1	1	g3001.t1
High	TLSDYNIQK	1	3	1	g1853.t1
High	LDEDVPR	1	1	1	g4628.t1
High	RDRDPYVGDIAVSGK	1	1	1	g5548.t1
High	AGDVALATAADSAWSAIK	1	1	1	g5090.t1
High	AGGAQVPAPGR	1	1	1	g3373.t1
High	RSVTVHAFEAQAR	1	1	1	g6826.t1
High	QTASWTVR	1	1	1	g9312.t1
High	NIHDFR	1	1	1	g6228.t1
High	EEILEAcR	1	1	1	g8452.t1

Modifications	ΔCn	q-Value	PEP	XCorr	Charge	MH+ [Da]	ΔM [ppm]
	####	0	3,7E-09	7,95	3	#######	5,52
	####	0	1,6E-10	7,89	3	#######	-0,14
	####	0	3,3E-10	7,87	2	#######	-0,17
	####	0	2,8E-10	7,85	3	#######	-0,76
M15(Oxidation); M16(Oxidation); M23(Oxidation); C31(Carbamidometh	####	0	1,5E-09	7,69	3	#######	0,69
	####	0	4,1E-10	7,65	3	#######	0,48
	####	0	1,2E-08	7,63	3	#######	0,92
M16(Oxidation)	####	0	2E-10	7,52	3	#######	-0,41
	####	0	1,2E-08	7,49	3	#######	-0,11
	####	0	8,3E-09	7,49	3	#######	-3,02
	####	0	3E-10	7,48	3	#######	0,72
M4(Oxidation); C26(Carbamidomethyl)	####	0	3,7E-11	7,45	3	#######	0,82
C21(Carbamidomethyl); C29(Carbamidomethyl)	####	0	5,6E-07	7,45	3	#######	0,13
C20(Carbamidomethyl)	####	0	2,8E-09	7,43	3	#######	0,37
M24(Oxidation)	####	0	4,3E-11	7,30	3	#######	-0,61
	####	0	5,9E-12	7,25	3	#######	-2,16
	####	0	1,8E-09	7,22	3	#######	1,63
M15(Oxidation)	####	0	9,2E-10	7,18	3	#######	-0,42
	####	0	1,8E-08	7,18	3	#######	-0,45
M16(Oxidation); M23(Oxidation); C31(Carbamidomethyl)	####	0	3,8E-07	7,17	3	#######	0,26
C18(Carbamidomethyl); C26(Carbamidomethyl)	####	0	5,7E-09	7,16	3	#######	-0,17
C16(Carbamidomethyl)	####	0	1,3E-08	7,07	3	#######	1,27
	####	0	5,7E-08	7,05	3	#######	-0,19
M26(Oxidation)	####	0	5,6E-12	7,04	3	#######	-0,44
C11(Carbamidomethyl)	####	0	2,1E-10	7,03	2	#######	0,08
M19(Oxidation)	####	0	1,9E-05	6,98	3	#######	3,14
M4(Oxidation)	####	0	1,9E-09	6,96	2	#######	-0,45
	####	0	1,7E-08	6,94	3	#######	-1,59
	####	0	4E-08	6,92	2	#######	1,33
	####	0	2E-10	6,91	3	#######	-0,67
M28(Oxidation)	####	0	6,8E-08	6,90	4	#######	4,24
M2(Oxidation); C15(Carbamidomethyl)	####	0	4,7E-11	6,87	2	#######	-0,87
M9(Oxidation)	####	0	1,3E-07	6,84	3	#######	-0,29
	####	0	5,2E-12	6,83	2	#######	0,98
	####	0	1,2E-05	6,82	3	#######	0,10
C26(Carbamidomethyl); C33(Carbamidomethyl)	####	0	5,4E-07	6,81	3	#######	1,83
	####	0	1,5E-08	6,79	3	#######	0,98
	####	0	2,5E-07	6,73	3	#######	1,36
	####	0	7E-11	6,71	3	#######	-0,75
	####	0	8,4E-11	6,70	2	#######	1,61
M26(Oxidation)	####	0	5,5E-07	6,70	3	#######	1,07
	####	0	1,4E-07	6,67	3	#######	-1,54
M13(Oxidation)	####	0	6,3E-13	6,67	3	#######	-1,35
	####	0	1,5E-11	6,66	2	#######	1,35
	####	0	5,7E-08	6,65	3	#######	-0,09
	####	0	5,8E-09	6,65	2	#######	-1,39
	####	0	3E-09	6,62	3	#######	0,47
	####	0	1,1E-08	6,61	3	#######	-1,27
M17(Oxidation)	####	0	3,4E-10	6,56	3	#######	-0,72
	####	0	5,9E-11	6,56	3	#######	0,74
C10(Carbamidomethyl)	####	0	1,1E-08	6,56	3	#######	1,56
	####	0	2,9E-09	6,54	3	#######	0,75
M7(Oxidation)	####	0	7,5E-08	6,53	3	#######	1,42
M2(Oxidation)	####	0	2,6E-08	6,52	3	#######	-0,18
	####	0	2,8E-08	6,49	2	#######	-1,24

	####	0	2,9E-09	6,47	2	#######	-0,42
C2(Carbamidomethyl)	####	0	3,8E-10	6,44	3	#######	-3,01
M3(Oxidation)	####	0	1,4E-06	6,42	3	#######	-0,19
	####	0	2,7E-08	6,42	2	#######	1,51
	####	0	2,1E-09	6,40	2	#######	1,31
	####	0	5,5E-08	6,39	3	#######	0,36
	####	0	4,4E-08	6,39	3	#######	1,25
C8(Carbamidomethyl); C14(Carbamidomethyl)	####	0	1,5E-07	6,39	2	#######	1,41
C2(Carbamidomethyl)	####	0	6,2E-09	6,38	3	#######	0,41
	####	0	1,9E-08	6,38	3	#######	0,19
C8(Carbamidomethyl); C24(Carbamidomethyl)	####	0	1,7E-06	6,37	3	#######	-1,51
	####	0	1,6E-07	6,37	2	#######	0,71
	####	0	1,5E-07	6,34	3	#######	-0,04
M2(Oxidation)	####	0	5,8E-10	6,32	3	#######	-0,47
	####	0	1,4E-07	6,31	3	#######	-0,20
	####	0	2,5E-09	6,31	3	#######	-0,58
	####	0	1E-08	6,30	3	#######	0,44
	####	0	1,6E-09	6,30	2	#######	0,41
	####	0	5,1E-08	6,29	3	#######	0,12
	####	0	2E-08	6,29	3	#######	0,22
C11(Carbamidomethyl)	####	0	8,8E-09	6,29	3	#######	-0,51
	####	0	2,1E-09	6,28	3	#######	-1,13
	####	0	4,3E-07	6,28	2	#######	-0,23
M12(Oxidation)	####	0	5,4E-09	6,28	3	#######	0,09
M10(Oxidation)	####	0	4,2E-07	6,27	3	#######	0,28
M3(Oxidation)	####	0	8E-09	6,27	2	#######	-1,01
M3(Oxidation)	####	0	1E-07	6,25	2	#######	0,23
M24(Oxidation)	####	0	9,7E-09	6,25	3	#######	-0,25
	####	0	3,2E-07	6,25	3	#######	-0,73
	####	0	2,3E-05	6,24	3	#######	-3,64
C19(Carbamidomethyl)	####	0	8,4E-10	6,23	2	#######	-0,28
	####	0	1,7E-06	6,22	2	#######	1,80
	####	0	5,8E-09	6,19	3	#######	3,59
M27(Oxidation); M31(Oxidation)	####	0	1,1E-08	6,18	3	#######	0,81
	####	0	3,5E-08	6,18	2	#######	-0,36
	####	0	9,9E-08	6,17	3	#######	-0,02
	####	0	3,7E-08	6,16	2	#######	0,97
	####	0	2,3E-09	6,15	2	#######	1,68
M5(Oxidation); C6(Carbamidomethyl); C23(Carbamidomethyl)	####	0	1,9E-07	6,15	3	#######	0,27
	####	0	1,9E-09	6,15	3	#######	-0,93
	####	0	1,3E-06	6,15	2	#######	0,30
	####	0	4,9E-09	6,13	3	#######	-2,12
	####	0	1E-10	6,13	3	#######	-0,04
	####	0	8,2E-06	6,12	3	#######	0,01
	####	0	1,2E-09	6,12	2	#######	-0,91
	####	0	1,1E-08	6,11	2	#######	1,61
	####	0	3E-10	6,11	3	#######	0,03
	####	0	8,1E-10	6,11	2	#######	-0,63
	####	0	1,8E-06	6,10	2	#######	-1,36
	####	0	3,8E-07	6,10	3	#######	-0,51
	####	0	5,3E-09	6,09	2	#######	-0,73
	####	0	2,3E-07	6,08	2	#######	-2,29
	####	0	3,2E-08	6,08	2	#######	-0,09
C10(Carbamidomethyl); C15(Carbamidomethyl)	####	0	5,8E-08	6,08	2	#######	0,79
	####	0	1,2E-09	6,05	2	#######	0,56
	####	0	1,3E-08	6,04	2	#######	-0,03
	####	0	1,1E-07	6,03	3	#######	-0,77
	####	0	5,9E-09	6,03	2	#######	0,27
	####	0	7,7E-11	6,03	3	#######	0,82

	####	0	1,8E-06	6,03	3	#######	0,60
C12(Carbamidomethyl)	####	0	1,6E-09	6,02	2	#######	-0,88
C4(Carbamidomethyl)	####	0	3,7E-08	6,02	3	#######	0,70
C11(Carbamidomethyl)	####	0	1,1E-07	6,01	2	#######	4,48
	####	0	4,1E-09	6,00	2	#######	0,41
	####	0	1,3E-06	5,98	3	#######	-1,04
C12(Carbamidomethyl)	####	0	9,8E-05	5,98	3	#######	1,39
C16(Carbamidomethyl); C21(Carbamidomethyl)	####	0	1,1E-05	5,97	3	#######	2,53
C10(Carbamidomethyl)	####	0	3E-08	5,97	2	#######	-0,72
	####	0	5,9E-09	5,96	2	#######	2,02
	####	0	8,9E-09	5,95	2	#######	-0,64
	####	0	3,9E-06	5,95	3	#######	-0,51
C8(Carbamidomethyl)	####	0	1,8E-07	5,92	3	#######	1,09
	####	0	1,2E-06	5,91	2	#######	-0,20
	####	0	4,3E-09	5,91	2	#######	0,01
	####	0	3,1E-10	5,90	3	#######	0,03
	####	0	1,4E-06	5,90	3	#######	-1,00
	####	0	3E-10	5,90	2	#######	-0,01
	####	0	1,6E-07	5,88	3	#######	-1,35
	####	0	2,9E-07	5,87	3	#######	2,16
M7(Oxidation)	####	0	7E-09	5,87	3	#######	0,41
	####	0	1,5E-08	5,86	2	#######	1,47
	####	0	2,2E-08	5,86	2	#######	1,38
	####	0	1,8E-07	5,85	3	#######	-2,93
C18(Carbamidomethyl); C26(Carbamidomethyl)	####	0	2,9E-05	5,83	3	#######	1,25
	####	0	4,5E-10	5,83	2	#######	-0,42
	####	0	1,9E-07	5,83	2	#######	-0,29
	####	0	2,6E-08	5,82	2	#######	0,08
	####	0	4,7E-08	5,82	2	#######	-1,07
	####	0	2E-07	5,82	3	#######	-6,95
	####	0	1,4E-09	5,82	2	#######	0,17
	####	0	1,7E-09	5,82	3	#######	-0,62
	####	0	4,2E-11	5,80	2	#######	-0,19
	####	0	2E-06	5,78	3	#######	0,83
C10(Carbamidomethyl); C17(Carbamidomethyl)	####	0	0,00048	5,78	3	#######	-5,10
M16(Oxidation)	####	0	4,2E-07	5,78	3	#######	-0,64
	####	0	2,3E-09	5,78	2	#######	0,47
M7(Oxidation)	####	0	1,8E-08	5,77	2	#######	-0,82
M7(Oxidation)	####	0	7E-08	5,76	2	#######	-0,04
M12(Oxidation)	####	0	1,3E-08	5,76	3	#######	-0,07
C26(Carbamidomethyl)	####	0	2,5E-09	5,76	3	#######	0,29
	####	0	5,5E-07	5,76	2	#######	1,04
	####	0	9,7E-10	5,74	2	#######	-1,57
	####	0	3,1E-09	5,73	2	#######	0,33
	####	0	4,7E-08	5,73	2	#######	-0,04
C12(Carbamidomethyl)	####	0	9E-09	5,73	3	#######	-0,72
	####	0	0,00081	5,72	3	#######	-2,14
	####	0	9,9E-07	5,70	3	#######	-0,20
	####	0	1,3E-05	5,70	3	#######	-0,78
	####	0	1,1E-07	5,69	2	#######	-0,50
	####	0	5,6E-07	5,68	3	#######	-2,88
	####	0	4,6E-08	5,68	2	#######	0,03
	####	0	4,5E-09	5,68	2	#######	-1,79
M21(Oxidation)	####	0	5,7E-06	5,68	3	#######	-0,41
	####	0	2,9E-07	5,67	3	#######	0,91
C8(Carbamidomethyl)	####	0	1,5E-07	5,67	2	#######	0,64
C8(Carbamidomethyl)	####	0	1,4E-08	5,67	2	#######	0,15
C3(Carbamidomethyl); C9(Carbamidomethyl)	####	0	4,8E-05	5,66	3	#######	0,25
	####	0	3,3E-10	5,65	2	#######	0,68

	####	0	1,3E-06	5,63	2	#######	6,82
	####	0	1,7E-07	5,63	2	#######	-1,06
C11(Carbamidomethyl)	####	0	1,7E-06	5,62	2	#######	1,15
C2(Carbamidomethyl); M27(Oxidation)	####	0	0,00019	5,62	3	#######	-0,98
	####	0	4E-08	5,62	3	#######	0,30
M8(Oxidation)	####	0	4E-07	5,61	3	#######	-0,37
	####	0	4,1E-05	5,60	3	#######	-1,19
	####	0	1,3E-07	5,60	2	#######	0,31
M3(Oxidation)	####	0	4,9E-06	5,60	3	#######	-1,83
C9(Carbamidomethyl)	####	0	2,2E-09	5,59	2	#######	-0,21
	####	0	2,4E-09	5,59	2	#######	-0,19
	####	0	2,1E-08	5,59	2	#######	1,70
	####	0	9,5E-08	5,59	3	#######	0,27
	####	0	2,6E-07	5,59	2	#######	-0,42
	####	0	1,7E-07	5,58	3	#######	-3,21
	####	0	1,5E-08	5,58	3	#######	-0,58
C1(Carbamidomethyl); M4(Oxidation); C8(Carbamidomethyl); C17(Carb	####	0	2,4E-05	5,57	3	#######	-2,04
	####	0	3,9E-07	5,56	2	#######	-0,55
	####	0	7,9E-08	5,55	3	#######	0,53
	####	0	7,5E-07	5,54	3	#######	-0,55
	####	0	1,4E-07	5,54	2	#######	0,44
	####	0	1,6E-07	5,52	2	#######	-1,49
	####	0	1,9E-06	5,51	2	#######	3,18
M2(Oxidation)	####	0	1,2E-08	5,51	2	#######	-0,32
C12(Carbamidomethyl)	####	0	2,3E-06	5,50	2	#######	2,45
C22(Carbamidomethyl); C30(Carbamidomethyl)	####	0	8,1E-07	5,50	4	#######	0,48
	####	0	8,8E-08	5,49	2	#######	1,09
M21(Oxidation); M22(Oxidation)	####	0	2,5E-05	5,47	3	#######	0,57
	####	0	2,4E-06	5,47	2	#######	-0,13
	####	0	8E-06	5,46	2	#######	-3,05
	####	0	2,4E-07	5,45	3	#######	-1,47
	####	0	2,7E-06	5,44	3	#######	0,82
	####	0	2,8E-05	5,43	3	#######	4,20
	####	0	0,00043	5,43	3	#######	-5,06
	####	0	2,8E-08	5,43	3	#######	0,18
	####	0	1,4E-07	5,42	2	#######	-0,08
	####	0	3,6E-05	5,42	3	#######	-0,60
C17(Carbamidomethyl)	####	0	4,2E-07	5,41	3	#######	0,37
M3(Oxidation)	####	0	1,3E-05	5,41	3	#######	1,38
M14(Oxidation)	####	0	4E-07	5,39	3	#######	-0,41
C5(Carbamidomethyl)	####	0	3,5E-09	5,39	2	#######	-0,15
C12(Carbamidomethyl)	####	0	1,5E-06	5,38	3	#######	1,43
	####	0	1,9E-08	5,38	3	#######	0,56
	####	0	0,0011	5,36	2	#######	0,83
	####	0	1,7E-08	5,36	3	#######	0,38
	####	0	5,5E-05	5,36	2	#######	-0,40
	####	0	1,2E-07	5,35	2	#######	1,46
	####	0	1,3E-07	5,35	2	#######	-1,64
C22(Carbamidomethyl)	####	0	8,4E-08	5,33	3	#######	-0,46
C8(Carbamidomethyl)	####	0	3,8E-06	5,33	2	#######	0,54
C5(Carbamidomethyl); C10(Carbamidomethyl)	####	0	2,2E-08	5,33	2	#######	1,19
C9(Carbamidomethyl)	####	0	2,9E-06	5,33	3	#######	0,58
	####	0	5,5E-08	5,32	2	#######	1,74
	####	0	1,4E-08	5,32	2	#######	-1,40
	####	0	3,6E-08	5,31	2	#######	-0,23
	####	0	3,4E-08	5,31	2	#######	-0,61
	####	0	1,3E-06	5,31	2	#######	-0,28
C3(Carbamidomethyl)	####	0	2,9E-09	5,31	2	#######	-0,13
M7(Oxidation)	####	0	1,8E-07	5,30	2	#######	-2,95

	####	0	7E-08	5,30	3	#######	-1,62
C9(Carbamidomethyl); C15(Carbamidomethyl)	####	0	0,00022	5,30	3	#######	-3,21
	####	0	2,4E-09	5,30	2	#######	-1,22
	####	0	0,00011	5,29	3	#######	-0,75
M13(Oxidation)	####	0	2,3E-05	5,28	3	#######	-0,43
M3(Oxidation)	####	0	3,1E-06	5,28	2	#######	-0,70
	####	0	6,1E-07	5,28	2	#######	-1,69
	####	0	6,8E-06	5,28	2	#######	-1,33
	####	0	2,5E-06	5,27	2	#######	0,41
C18(Carbamidomethyl)	####	0	3,1E-08	5,27	3	#######	-1,76
C25(Carbamidomethyl)	####	0	2,8E-06	5,26	3	#######	0,60
	####	0	1,1E-06	5,25	2	#######	-0,69
C12(Carbamidomethyl)	####	0	7,2E-06	5,25	2	#######	0,48
M10(Oxidation)	####	0	7,3E-05	5,25	3	#######	-1,21
	####	0	5,5E-07	5,25	3	#######	0,43
	####	0	8,4E-06	5,24	3	#######	-1,27
M18(Oxidation); M22(Oxidation)	####	0	4,5E-05	5,23	3	#######	1,07
	####	0	7,1E-07	5,23	2	#######	-1,44
C18(Carbamidomethyl)	####	0	2,2E-05	5,23	3	#######	-4,56
	####	0	5,4E-08	5,22	3	#######	-0,50
	####	0	9,7E-08	5,22	3	#######	0,06
	####	0	0,00058	5,22	2	#######	-2,73
	####	0	1,1E-07	5,21	2	#######	0,12
C14(Carbamidomethyl)	####	0	1,7E-07	5,21	2	#######	-0,14
	####	0	2,2E-06	5,21	3	#######	0,82
	####	0	4,7E-09	5,21	2	#######	-0,20
	####	0	3,3E-06	5,21	2	#######	-2,04
C17(Carbamidomethyl)	####	0	1,6E-05	5,21	3	#######	-0,90
C8(Carbamidomethyl)	####	0	1,4E-07	5,20	2	#######	-0,62
C13(Carbamidomethyl)	####	0	3,2E-08	5,20	2	#######	-0,88
	####	0	1E-06	5,20	2	#######	-0,75
M5(Oxidation)	####	0	5,8E-05	5,19	3	#######	-0,75
C13(Carbamidomethyl)	####	0	2,8E-05	5,19	3	#######	1,57
	####	0	1,8E-07	5,19	3	#######	5,19
	####	0	1,1E-08	5,19	3	#######	0,14
	####	0	1,1E-05	5,18	3	#######	-1,04
	####	0	2E-08	5,18	2	#######	0,02
	####	0	2,4E-08	5,18	2	#######	-0,35
C5(Carbamidomethyl)	####	0	2,3E-06	5,17	2	#######	-1,70
	####	0	1,3E-07	5,17	3	#######	1,07
C13(Carbamidomethyl)	####	0	3,4E-05	5,15	3	#######	0,80
	####	0	1,9E-07	5,15	2	#######	-0,39
C9(Carbamidomethyl)	####	0	1,3E-07	5,15	2	#######	-0,27
	####	0	2,4E-06	5,14	3	#######	0,12
	####	0	9,6E-05	5,14	3	#######	-4,86
	####	0	1,2E-05	5,14	2	#######	-2,23
	####	0	4,6E-09	5,13	2	#######	-0,16
	####	0	1,1E-05	5,13	2	#######	0,40
M13(Oxidation)	####	0	2,7E-05	5,12	3	#######	1,46
C7(Carbamidomethyl)	####	0	4,3E-06	5,12	3	#######	-1,03
	####	0	6,1E-07	5,12	2	#######	-0,63
	####	0	5,3E-07	5,12	2	#######	-0,52
M14(Oxidation)	####	0	1,7E-05	5,10	3	#######	1,91
	####	0	6,9E-08	5,10	2	#######	0,34
	####	0	3,7E-05	5,09	2	#######	3,54
	####	0	1,2E-07	5,08	3	#######	-1,05
	####	0	2,4E-06	5,08	2	#######	-0,65
	####	0	1,1E-06	5,08	3	#######	-0,80
	####	0	1,6E-06	5,08	3	#######	-0,70

C14(Carbamidomethyl)	####	0	8,8E-06	5,07	2	#######	0,25
M1(Oxidation); M7(Oxidation)	####	0	0,00066	5,07	3	#######	-2,27
	####	0	3,1E-09	5,07	4	#######	-0,17
	####	0	4,1E-06	5,06	2	#######	-0,18
	####	0	1,6E-07	5,06	2	#######	1,00
C8(Carbamidomethyl)	####	0	1,4E-08	5,05	2	#######	1,32
	####	0	1,6E-06	5,05	2	#######	2,24
	####	0	1,7E-07	5,03	2	#######	-0,98
	####	0	1E-05	5,03	3	#######	-0,92
	####	0	1,9E-06	5,03	3	#######	-0,09
	####	0	1,5E-07	5,02	2	#######	0,02
	####	0	8,6E-06	5,02	3	#######	3,94
	####	0	1,4E-05	5,02	4	#######	-0,98
	####	0	3,6E-07	5,00	2	#######	-0,27
	####	0	6,4E-06	5,00	3	#######	-0,01
C9(Carbamidomethyl); M11(Oxidation)	####	0	2,8E-05	4,99	3	#######	-1,12
C22(Carbamidomethyl)	####	0	7,3E-08	4,99	3	#######	0,39
	####	0	6,6E-06	4,97	3	#######	-1,69
	####	0	1E-07	4,97	2	#######	-0,28
M16(Oxidation)	####	0	4,5E-08	4,97	2	#######	0,63
	####	0	1,7E-07	4,96	2	#######	-2,07
	####	0	5,3E-09	4,96	2	#######	2,16
C9(Carbamidomethyl)	####	0	9,6E-08	4,95	3	#######	1,03
	####	0	6,9E-07	4,94	3	#######	-6,05
	####	0	1,6E-07	4,93	3	#######	-1,13
	####	0	3,5E-06	4,93	2	#######	-0,60
M5(Oxidation)	####	0	0,00038	4,92	3	#######	-1,68
M4(Oxidation)	####	0	1,7E-07	4,92	2	#######	0,18
	####	0	1,6E-06	4,92	3	#######	-0,28
	####	0	2,4E-07	4,92	2	#######	-2,17
M3(Oxidation)	####	0	8E-05	4,92	2	#######	0,51
M3(Oxidation); M7(Oxidation); C9(Carbamidomethyl)	####	0	1,3E-08	4,92	3	#######	0,37
	####	0	1,9E-06	4,91	2	#######	-0,72
	####	0	8,6E-07	4,91	2	#######	-3,53
	####	0	2,1E-06	4,91	2	#######	-0,79
	####	0	2,4E-08	4,90	2	#######	1,61
M16(Oxidation)	####	0	5,6E-07	4,89	2	#######	0,72
M7(Oxidation)	####	0	0,0001	4,88	2	#######	-0,25
	####	0	2,4E-06	4,88	2	#######	0,53
	####	0	2,6E-07	4,88	3	#######	0,30
M19(Oxidation); C29(Carbamidomethyl)	####	0	4,4E-05	4,87	4	#######	1,05
	####	0	1,5E-06	4,87	2	#######	0,14
	####	0	0,00027	4,86	3	#######	-1,12
	####	0	7,6E-05	4,86	3	#######	-0,05
	####	0	1,1E-05	4,85	3	#######	0,14
	####	0	0,00033	4,85	3	#######	0,11
	####	0	4,5E-08	4,84	3	#######	0,89
	####	0	0,00036	4,84	2	#######	-1,04
	####	0	8,5E-06	4,83	2	#######	-0,76
	####	0	6,2E-06	4,83	2	#######	0,50
	####	0	2,6E-08	4,83	2	#######	-0,25
	####	0	1,8E-08	4,81	3	#######	-0,05
	####	0	3,3E-05	4,81	3	#######	2,66
	####	0	1E-05	4,81	2	#######	-0,70
	####	0	0,00014	4,81	3	#######	-0,79
C8(Carbamidomethyl)	####	0	1,1E-07	4,80	2	#######	-2,18
	####	0	2,7E-07	4,80	2	#######	-0,46
	####	0	7,5E-08	4,80	2	#######	-2,21
	####	0	0,00029	4,79	3	#######	0,01

	####	0	1,2E-05	4,78	2	#######	0,33
M3(Oxidation); C16(Carbamidomethyl)	####	0	4,9E-07	4,78	3	#######	0,36
C8(Carbamidomethyl); C14(Carbamidomethyl)	####	0	1,7E-07	4,78	2	#######	-0,07
	####	0	3,4E-06	4,77	2	#######	0,07
	####	0	2,1E-06	4,76	2	#######	-0,13
	####	0	1,3E-05	4,76	3	#######	-1,57
	####	0	5,5E-07	4,76	2	#######	-0,58
	####	0	7,1E-09	4,75	3	#######	-0,81
	####	0	1,4E-06	4,75	3	#######	-0,46
	####	0	4,1E-07	4,74	2	#######	0,99
M4(Oxidation)	####	0	2,1E-08	4,74	3	#######	-0,23
	####	0	6,4E-06	4,74	2	#######	-0,92
	####	0	5,6E-05	4,73	2	#######	0,46
	####	0	2,6E-06	4,73	3	#######	-0,15
C10(Carbamidomethyl); C14(Carbamidomethyl)	####	0	5,6E-07	4,73	2	#######	-0,09
	####	0	0,00041	4,73	3	#######	-0,15
C2(Carbamidomethyl)	####	0	4,4E-08	4,72	2	#######	1,15
	####	0	1,8E-06	4,72	2	#######	-0,39
M10(Oxidation)	####	0	9,5E-07	4,72	2	#######	-2,05
	####	0	8,1E-08	4,71	2	#######	0,97
C6(Carbamidomethyl); C13(Carbamidomethyl)	####	0	1,3E-06	4,70	2	#######	-1,02
	####	0	7,4E-07	4,70	3	#######	0,19
	####	0	7,5E-06	4,70	3	#######	-0,57
	####	0	1,4E-05	4,69	2	#######	-1,12
	####	0	6,5E-07	4,69	2	#######	-0,04
C29(Carbamidomethyl)	####	0	4,5E-05	4,69	4	#######	0,79
M6(Oxidation)	####	0	8,8E-07	4,68	2	#######	-0,39
	####	0	6,3E-05	4,67	2	#######	-1,29
	####	0	1,8E-07	4,67	2	#######	-0,34
C7(Carbamidomethyl)	####	0	1,3E-06	4,66	2	#######	0,78
	####	0	8,6E-05	4,66	2	#######	-1,88
	####	0	3,8E-05	4,65	2	#######	-0,20
M3(Oxidation)	####	0	5,8E-06	4,65	3	#######	1,04
	####	0	0,00078	4,65	2	#######	-0,94
M7(Oxidation)	####	0	1,2E-06	4,65	3	#######	-0,29
	####	0	1,6E-06	4,64	3	#######	-0,18
	####	0	3,2E-06	4,64	2	#######	-0,57
	####	0	1,9E-07	4,63	2	#######	-1,33
	####	0	2,5E-06	4,63	2	#######	-1,33
M12(Oxidation); M14(Oxidation)	####	0	7,9E-08	4,63	2	#######	-0,91
	####	0	1,1E-05	4,62	2	#######	1,60
	####	0	1,4E-05	4,62	2	#######	0,25
	####	0	4,5E-07	4,62	3	#######	-1,38
	####	0	0,00085	4,60	3	#######	-0,75
	####	0	1,7E-05	4,60	2	#######	-2,94
	####	0	5,4E-05	4,60	2	#######	-0,09
	####	0	0,00017	4,60	2	#######	-1,41
	####	0	2E-07	4,59	2	#######	0,11
	####	0	1,8E-07	4,59	2	#######	-0,24
	####	0	0,00079	4,59	3	#######	3,16
C1(Carbamidomethyl)	####	0	3,8E-05	4,58	3	#######	0,54
	####	0	0,0008	4,58	3	#######	3,18
	####	0	4,4E-07	4,57	2	#######	-1,87
	####	0	2,4E-06	4,57	3	#######	1,79
M6(Oxidation)	####	0	2E-05	4,57	2	#######	-0,55
	####	0	1,6E-07	4,56	3	#######	-0,14
M18(Oxidation)	####	0	1E-06	4,56	2	#######	-0,21
	####	0	1,1E-07	4,56	2	#######	-0,85
	####	0	0,00011	4,55	2	#######	2,04

	####	0	4,6E-06	4,55	3	#######	-0,51
	####	0	5,9E-07	4,54	2	#######	-0,52
M7(Oxidation)	####	0	0,00011	4,54	3	#######	-0,73
	####	0	7E-06	4,53	3	#######	0,13
M2(Oxidation); M7(Oxidation)	####	0	9,4E-06	4,53	2	#######	-0,67
	####	0	6,5E-07	4,53	2	#######	-0,19
M11(Oxidation)	####	0	1,8E-06	4,53	2	#######	-0,32
C4(Carbamidomethyl); C18(Carbamidomethyl)	####	0	5,6E-05	4,52	3	#######	-0,28
	####	0	0,00011	4,52	3	#######	1,62
M1(Oxidation); M7(Oxidation)	####	0	5,4E-05	4,52	3	#######	-0,18
	####	0	5,9E-05	4,52	3	#######	0,57
	####	0	8,2E-05	4,51	3	#######	-0,33
	####	0	4,2E-05	4,51	3	#######	1,33
	####	0	4,5E-06	4,51	3	#######	-0,02
	####	0	3,7E-05	4,50	3	#######	2,06
	####	0	2,3E-06	4,50	3	#######	3,13
	####	0	9,2E-05	4,49	2	#######	-0,45
M15(Oxidation)	####	0	3,9E-07	4,49	2	#######	2,48
	####	0	1,8E-05	4,49	2	#######	-0,74
	####	0	0,00062	4,49	3	#######	-2,22
	####	0	7,4E-06	4,48	2	#######	-0,49
	####	0	7,9E-06	4,46	2	#######	-0,72
	####	0	0,00052	4,46	3	#######	-0,26
	####	0	5,7E-06	4,46	2	#######	1,94
	####	0	4,6E-05	4,45	3	#######	-4,24
C2(Carbamidomethyl); C11(Carbamidomethyl); M16(Oxidation)	####	0	4E-06	4,45	3	#######	0,52
	####	0	7,4E-07	4,45	3	#######	0,32
	####	0	1,6E-05	4,45	2	#######	-1,21
	####	0	6E-06	4,45	2	#######	0,03
	####	0	2,1E-05	4,44	2	#######	-0,16
	####	0	0,00077	4,44	2	#######	2,10
	####	0	2,2E-05	4,43	2	#######	-0,41
	####	0	5,2E-06	4,42	4	#######	-0,67
	####	0	6,3E-05	4,42	2	#######	0,00
	####	0	1E-06	4,39	2	#######	0,96
M5(Oxidation); M8(Oxidation)	####	0	1,9E-06	4,39	3	#######	-0,92
	####	0	0,00014	4,39	2	#######	-0,60
	####	0	0,00026	4,38	2	#######	-1,46
	####	0	3,5E-06	4,38	2	#######	-0,81
	####	0	8,9E-07	4,38	2	#######	-0,54
	####	0	8,8E-06	4,36	3	#######	0,87
	####	0	4,6E-05	4,35	2	#######	-0,23
	####	0	0,0001	4,35	3	#######	-1,37
	####	0	6,4E-07	4,35	2	#######	-0,40
M3(Oxidation)	####	0	6,3E-05	4,34	2	#######	-0,04
C4(Carbamidomethyl)	####	0	1,2E-05	4,33	2	#######	-0,97
	####	0	1E-05	4,33	2	#######	-0,05
	####	0	2,9E-05	4,32	2	#######	0,68
	####	0	2,7E-05	4,32	2	#######	-0,36
C3(Carbamidomethyl); C19(Carbamidomethyl)	####	0	0,00146	4,32	3	#######	-1,76
	####	0	3,5E-07	4,31	2	#######	-0,24
M3(Oxidation)	####	0	6,2E-06	4,31	2	#######	-0,06
	####	0	0,00015	4,31	2	#######	-0,07
C2(Carbamidomethyl)	####	0	0,00148	4,31	2	#######	-2,68
	####	0	6,9E-06	4,31	2	#######	-0,57
	####	0	3,7E-06	4,30	3	#######	-0,07
	####	0	0,00049	4,30	3	#######	-0,32
	####	0	3,5E-08	4,29	3	#######	-0,28
	####	0	0,00072	4,29	3	#######	0,72

	####	0	3,1E-06	4,29	2	#######	-1,07
M13(Oxidation)	####	0	1,1E-06	4,29	3	#######	-0,93
M8(Oxidation)	####	0	0,00011	4,28	3	#######	-0,17
	####	0	1,5E-05	4,28	2	#######	-0,96
	####	0	0,00014	4,28	2	#######	-1,02
C7(Carbamidomethyl)	####	0	2,3E-07	4,28	2	#######	-0,29
	####	0	1,7E-05	4,28	2	#######	-2,46
	####	0	1,5E-06	4,27	3	#######	-1,06
C3(Carbamidomethyl)	####	0	2,2E-05	4,27	3	#######	0,54
	####	0	2E-05	4,27	3	#######	-0,62
	####	0	8,9E-06	4,27	2	#######	1,06
	####	0	2,9E-06	4,26	2	#######	0,39
	####	0	1,3E-06	4,25	2	#######	-0,29
	####	0	4,4E-05	4,25	2	#######	-2,03
	####	0	1E-06	4,24	3	#######	-0,32
	####	0	0,00016	4,24	2	#######	-0,18
C10(Carbamidomethyl)	####	0	3,2E-05	4,23	2	#######	1,04
	####	0	2,4E-06	4,23	2	#######	-0,68
	####	0	0,00029	4,23	2	#######	-0,23
C4(Carbamidomethyl)	####	0	0,00016	4,23	2	#######	-0,51
	####	0	2,7E-05	4,22	2	#######	-0,71
	####	0	2,8E-05	4,21	2	#######	-0,47
	####	0	7,3E-06	4,21	2	#######	-0,52
C3(Carbamidomethyl)	####	0	6E-06	4,20	3	#######	0,04
	####	0	8,4E-05	4,19	3	#######	1,71
C17(Carbamidomethyl)	####	0	1,2E-06	4,19	3	#######	0,66
	####	0	0,00101	4,18	3	#######	-1,28
M10(Oxidation)	####	0	1,1E-06	4,18	2	#######	-0,24
	####	0	0,00022	4,18	3	#######	0,51
	####	0	2,2E-06	4,18	2	#######	-0,94
	####	0	0,00017	4,17	2	#######	0,34
	####	0	0,0005	4,17	2	#######	-1,16
	####	0	6,1E-05	4,17	2	#######	1,25
	####	0	4,8E-06	4,16	2	#######	-0,02
M12(Oxidation); M16(Oxidation)	####	0	4,1E-05	4,16	3	#######	-0,32
	####	0	6,9E-05	4,16	2	#######	-1,24
	####	0	4,4E-05	4,16	3	#######	1,26
	####	0	0,00023	4,16	2	#######	-2,78
	####	0	7,2E-05	4,16	2	#######	-0,28
	####	0	8,9E-05	4,15	2	#######	-1,55
M1(Oxidation)	####	0	2,4E-05	4,15	4	#######	2,74
	####	0	0,0006	4,15	2	#######	-1,74
	####	0	0,00176	4,15	3	#######	-1,09
	####	0	1,7E-05	4,14	2	#######	-0,48
	####	0	3,6E-06	4,14	2	#######	-0,84
	####	0	0,00056	4,14	3	#######	3,74
C3(Carbamidomethyl); M4(Oxidation)	####	0	5,2E-05	4,14	2	#######	-0,88
	####	0	0,00019	4,13	3	#######	5,82
	####	0	0,00028	4,13	2	#######	0,74
	####	0	3,6E-07	4,13	2	#######	-0,04
	####	0	4,5E-05	4,13	2	#######	-0,16
	####	0	6,7E-06	4,12	2	#######	-0,37
	####	0	5,4E-06	4,12	2	#######	-0,44
	####	0	4,5E-06	4,12	2	#######	1,05
	####	0	0,00051	4,11	2	#######	-0,48
C6(Carbamidomethyl)	####	0	3,8E-05	4,11	2	#######	-0,08
	####	0	0,00018	4,11	2	#######	0,17
	####	0	0,00515	4,10	3	#######	-0,47
	####	0	1,2E-06	4,10	2	#######	-0,16

	####	0	6,6E-06	4,10	2	#######	0,08
	####	0	3,4E-05	4,10	2	#######	-0,27
	####	0	1,3E-05	4,09	3	#######	1,24
M7(Oxidation); M8(Oxidation)	####	0	2,4E-05	4,08	2	#######	0,20
	####	0	4,7E-05	4,07	2	#######	0,67
	####	0	0,00016	4,07	2	#######	-0,62
M3(Oxidation); C4(Carbamidomethyl)	####	0	7,2E-05	4,07	3	#######	-1,50
	####	0	0,00033	4,07	2	#######	-1,32
C11(Carbamidomethyl)	####	0	3,1E-05	4,07	2	#######	-0,33
	####	0	3,2E-05	4,07	2	#######	0,15
	####	0	2,8E-05	4,06	3	#######	1,47
	####	0	9,9E-07	4,05	3	#######	0,03
	####	0	0,00037	4,05	2	#######	-0,39
	####	0	0,00078	4,05	2	#######	-0,41
	####	0	6,5E-05	4,05	2	#######	0,71
	####	0	0,00066	4,03	2	#######	-0,55
C11(Carbamidomethyl)	####	0	0,00044	4,02	3	#######	-0,38
	####	0	1,8E-06	4,02	2	#######	-0,39
	####	0	1,3E-05	4,02	2	#######	-0,85
	####	0	0,00155	4,02	3	#######	-0,13
M1(Oxidation)	####	0	6,3E-06	4,01	2	#######	-0,83
	####	0	0,00025	4,00	3	#######	-0,30
	####	0	1,6E-05	4,00	2	#######	-1,60
	####	0	2,3E-05	4,00	2	#######	-2,03
	####	0	1,9E-05	3,99	2	#######	-0,12
	####	0	1,2E-05	3,97	2	#######	0,42
	####	0	0,00019	3,97	2	#######	1,11
M7(Oxidation)	####	0	0,00064	3,96	2	#######	0,23
	####	0	1,5E-05	3,96	2	#######	0,94
	####	0	7,1E-06	3,95	2	#######	-0,85
	####	0	0,00071	3,95	2	#######	-0,57
	####	0	3,9E-05	3,94	2	#######	0,01
	####	0	5,1E-05	3,94	2	#######	0,50
	####	0	0,00021	3,94	2	#######	-0,97
	####	0	1E-04	3,93	2	#######	1,34
	####	0	0,00061	3,93	2	#######	-0,69
	####	0	2,8E-05	3,93	3	#######	-0,10
	####	0	3E-06	3,93	2	#######	-0,16
	####	0	0,00076	3,93	2	#######	-0,50
	####	0	0,00042	3,92	2	#######	-0,31
	####	0	6,7E-05	3,92	2	#######	-1,75
	####	0	2,8E-05	3,92	2	#######	-0,45
C3(Carbamidomethyl)	####	0	3,9E-05	3,91	2	#######	-0,41
M3(Oxidation)	####	0	0,0002	3,91	2	#######	-0,32
	####	0	0,00039	3,91	2	#######	-2,97
	####	0	6,3E-05	3,90	2	#######	-0,52
	####	0	6,9E-06	3,90	2	#######	-0,82
	####	0	3,7E-05	3,89	4	#######	-0,30
M14(Oxidation)	####	0	4,6E-05	3,89	3	#######	1,10
	####	0	9,3E-05	3,89	2	#######	-0,08
C3(Carbamidomethyl)	####	0	4,1E-05	3,89	2	#######	-0,43
	####	0	1,3E-05	3,89	2	#######	-0,44
	####	0	8,3E-05	3,88	2	#######	-0,06
M5(Oxidation); C7(Carbamidomethyl)	####	0	0,00018	3,87	2	#######	-0,84
	####	0	0,00022	3,87	2	#######	-1,50
	####	0	0,00023	3,87	3	#######	-0,76
	####	0	1,9E-05	3,86	2	#######	-0,82
M10(Oxidation)	####	0	3E-05	3,86	2	#######	-0,42
	####	0	0,00019	3,86	3	#######	0,50

	####	0	8,8E-05	3,85	2	#######	-0,57
M3(Oxidation); M7(Oxidation); C8(Carbamidomethyl)	####	0	0,00013	3,85	2	#######	-1,44
M9(Oxidation)	####	0	9,9E-06	3,85	3	#######	0,19
	####	0	7,7E-07	3,85	3	#######	0,17
	####	0	7E-06	3,83	2	#######	-0,41
	####	0	2,6E-05	3,83	3	#######	-1,12
	####	0	6,5E-05	3,83	2	#######	-1,05
	####	0	1,1E-06	3,83	2	#######	-0,37
	####	0	6,7E-07	3,82	3	#######	0,49
M3(Oxidation)	####	0	2,8E-05	3,82	3	#######	0,04
	####	0	7,7E-05	3,82	2	#######	0,45
	####	0	0,00172	3,82	2	#######	-0,13
M11(Oxidation)	####	0	0,0003	3,82	3	#######	3,81
	####	0	0,00075	3,81	2	#######	-1,32
	####	0	0,00016	3,81	2	#######	-1,61
	####	0	0,00021	3,81	2	#######	-0,93
M3(Oxidation)	####	0	1E-05	3,81	3	#######	-0,21
	####	0	6,9E-05	3,80	2	#######	-0,64
	####	0	5,3E-05	3,80	2	#######	0,17
	####	0	0,00015	3,80	2	#######	0,95
C19(Carbamidomethyl)	####	0	0,0008	3,79	3	#######	2,49
	####	0	1,3E-06	3,78	3	#######	-1,80
	####	0	1,1E-05	3,78	2	#######	-0,17
	####	0	0,0048	3,78	2	#######	-0,11
M10(Oxidation)	####	0	0,00163	3,77	3	#######	-5,82
C15(Carbamidomethyl)	####	0	4,7E-05	3,77	3	#######	-0,43
	####	0	0,00093	3,77	2	#######	-0,70
	####	0	4,7E-09	3,76	3	#######	-0,52
	####	0	0,00282	3,76	2	#######	-1,57
	####	0	1,9E-06	3,76	2	#######	0,02
	####	0	0,00453	3,76	3	#######	-1,16
	####	0	0,00014	3,76	2	#######	0,29
	####	0	5,4E-05	3,76	2	#######	0,44
	####	0	0,0001	3,76	2	#######	0,21
	####	0	9,1E-05	3,75	2	#######	-2,47
	####	0	0,00266	3,75	2	#######	-1,35
	####	0	0,00013	3,75	2	#######	-0,31
	####	0	0,0001	3,75	2	#######	-0,51
	####	0	0,00012	3,74	2	#######	-0,73
	####	0	0,00011	3,74	2	#######	-0,23
	####	0	0,0031	3,74	3	#######	2,06
	####	0	0,0005	3,73	2	#######	-0,59
C18(Carbamidomethyl)	####	0	6,2E-06	3,73	3	#######	-0,67
	####	0	1,9E-05	3,73	2	#######	0,37
M9(Oxidation)	####	0	0,00014	3,72	2	#######	-0,04
	####	0	1,8E-06	3,72	3	#######	-0,04
	####	0	0,00029	3,72	2	#######	-2,21
	####	0	0,00344	3,72	3	#######	0,63
	####	0	0,00011	3,71	2	976,44756	-0,81
	####	0	0,00011	3,71	2	#######	-0,37
	####	0	0,00017	3,70	2	#######	-1,14
C7(Carbamidomethyl)	####	0	9,8E-05	3,70	2	#######	-0,54
C4(Carbamidomethyl)	####	0	5,4E-05	3,70	3	#######	0,73
	####	0	5,1E-05	3,70	2	#######	-0,50
	####	0	0,00091	3,69	2	#######	-1,81
	####	0	0,00012	3,69	2	#######	-1,09
	####	0	4,9E-05	3,69	2	#######	-0,12
M12(Oxidation)	####	0	1,4E-05	3,67	4	#######	1,46
	####	0	5,3E-05	3,67	2	#######	0,19

	####	0	0,00197	3,66	2	#######	-0,10
	####	0	0,0002	3,66	2	#######	-1,57
	####	0	3,8E-05	3,66	2	#######	1,02
	####	0	8,8E-05	3,66	2	#######	0,09
	####	0	3,5E-05	3,65	2	#######	1,22
	####	0	0,0025	3,65	2	#######	-0,29
M11(Oxidation)	####	0	1,8E-05	3,65	3	#######	0,82
M6(Oxidation)	####	0	0,00021	3,65	2	#######	0,97
	####	0	4,7E-05	3,65	2	#######	-0,56
	####	0	2,2E-05	3,64	2	#######	0,03
	####	0	0,00273	3,64	2	#######	0,35
	####	0	0,00153	3,64	2	#######	-0,63
	####	0	1,9E-06	3,64	3	#######	-0,92
	####	0	0,00501	3,63	2	#######	-1,15
	####	0	0,00041	3,63	2	#######	-1,32
	####	0	0,00014	3,63	2	#######	-0,01
	####	0	5,8E-05	3,63	2	#######	0,04
	####	0	1E-04	3,63	2	#######	-1,89
	####	0	0,00015	3,62	2	#######	0,24
	####	0	3E-05	3,62	2	#######	-1,46
C1(Carbamidomethyl)	####	0	2,1E-05	3,60	3	#######	0,40
	####	0	0,00052	3,60	3	#######	0,69
	####	0	0,00322	3,60	2	#######	-0,19
	####	0	2,8E-05	3,60	2	#######	-0,81
	####	0	0,00038	3,59	3	#######	0,21
M4(Oxidation)	####	0	0,00039	3,59	2	#######	-2,02
	####	0	0,00042	3,59	2	#######	-0,07
M24(Oxidation); M26(Oxidation)	####	0	0,00159	3,59	4	#######	-0,58
	####	0	2,8E-05	3,58	2	#######	0,51
	####	0	0,00064	3,57	2	#######	0,82
	####	0	0,00516	3,57	2	#######	-0,29
M2(Oxidation)	####	0	2,3E-05	3,56	3	#######	-0,37
	####	0	0,00023	3,56	4	#######	-0,11
	####	0	4,2E-05	3,55	3	#######	-0,41
	####	0	0,00062	3,55	3	#######	-0,98
	####	0	0,00503	3,55	2	#######	-0,88
	####	0	0,00176	3,53	2	#######	-0,44
	####	0	3,9E-06	3,53	3	#######	-0,08
	####	0	0,00097	3,53	3	#######	-1,06
	####	0	0,0001	3,53	2	#######	-0,38
	####	0	0,00252	3,53	3	#######	-0,18
	####	0	0,00165	3,52	2	#######	-0,50
	####	0	0,00071	3,52	2	#######	-1,87
M9(Oxidation)	####	0	0,00015	3,51	2	#######	0,70
C7(Carbamidomethyl)	####	0	0,00017	3,50	2	#######	0,36
	####	0	0,00022	3,50	2	#######	-1,56
M16(Oxidation)	####	0	5,4E-06	3,50	4	#######	-0,37
	####	0	0,00013	3,50	2	988,54167	-0,55
	####	0	0,00072	3,49	2	#######	0,58
C11(Carbamidomethyl)	####	0	4,9E-05	3,49	2	#######	-0,76
	####	0	7,8E-05	3,48	2	#######	0,49
	####	0	9,4E-05	3,48	4	#######	-0,21
	####	0	0,00067	3,47	3	#######	-0,97
C1(Carbamidomethyl)	####	0	0,00019	3,47	2	#######	0,25
	####	0	0,00016	3,47	2	#######	1,16
	####	0	0,00338	3,47	2	#######	2,48
	####	0	0,00057	3,47	2	#######	-1,06
	####	0	1,8E-05	3,46	2	#######	-1,28
	####	0	3,3E-06	3,44	3	#######	-0,77

	####	0	6,9E-05	3,44	2	#######	-0,65
M13(Oxidation)	####	0	0,00346	3,44	2	#######	-1,27
	####	0	0,00015	3,44	2	#######	-0,38
	####	0	2,2E-06	3,43	3	#######	-0,57
	####	0	9,8E-05	3,43	3	#######	-2,40
	####	0	0,00508	3,42	2	987,54656	-0,44
C7(Carbamidomethyl)	####	0	0,00022	3,42	2	#######	-0,32
	####	0	0,0001	3,41	2	#######	1,29
	####	0	0,00131	3,41	2	#######	-1,62
	####	0	0,00325	3,40	2	#######	-0,36
	####	0	0,00027	3,40	2	#######	-0,74
	####	0	6E-05	3,40	3	#######	0,41
	####	0	0,00188	3,40	2	#######	-0,51
	####	0	7E-05	3,39	2	#######	-0,05
	####	0	0,00129	3,38	2	#######	-0,93
	####	0	4,2E-05	3,38	2	#######	0,27
	####	0	0,00013	3,38	3	#######	-1,95
	####	0	0,00353	3,37	2	#######	-0,96
	####	0	0,00021	3,37	2	#######	0,07
	####	0	0,0003	3,37	2	975,51042	-0,18
M29(Oxidation)	####	0	1,1E-05	3,36	5	#######	-0,60
	####	0	0,00114	3,35	3	#######	-0,10
	####	0	0,00107	3,35	2	#######	-0,40
	####	0	0,00208	3,35	2	#######	-1,11
	####	0	0,00074	3,35	2	#######	0,38
	####	0	4,4E-05	3,34	3	#######	-0,90
	####	0	4,1E-05	3,34	2	#######	-1,24
	####	0	2,9E-05	3,34	2	#######	-0,10
	####	0	0,0002	3,33	2	#######	-0,73
	####	0	0,00179	3,33	3	#######	-0,33
	####	0	0,00067	3,33	3	#######	-1,74
	####	0	0,00038	3,33	2	#######	2,39
	####	0	5,2E-05	3,33	2	#######	-0,47
	####	0	4E-05	3,32	2	#######	-0,78
	####	0	0,00062	3,32	2	#######	0,03
	####	0	0,00081	3,31	2	#######	-0,59
	####	0	0,00114	3,30	2	#######	-1,42
	####	0	0,00259	3,29	2	#######	1,12
	####	0	0,00052	3,29	2	#######	-0,34
	####	0	0,00432	3,29	2	#######	-2,52
M8(Oxidation)	####	0	0,00286	3,28	2	#######	-4,45
	####	0	0,0028	3,28	2	935,49498	0,46
	####	0	2,4E-05	3,28	2	#######	-0,10
	####	0	0,00014	3,26	2	#######	0,86
	####	0	0,00368	3,26	2	#######	-1,13
	####	0	0,00094	3,26	2	#######	-0,07
	####	0	0,00591	3,25	2	#######	-0,20
	####	0	0,00027	3,24	2	#######	-0,54
	####	0	3E-05	3,24	2	#######	-0,34
	####	0	0,00012	3,24	2	#######	-0,40
	####	0	0,00559	3,24	2	#######	-0,17
	####	0	0,0014	3,24	3	#######	-0,27
	####	0	0,00072	3,23	2	#######	-0,92
	####	0	0,00038	3,23	2	#######	0,11
	####	0	0,00044	3,23	2	#######	1,61
	####	0	0,0005	3,22	2	#######	0,22
	####	0	0,00516	3,22	3	#######	-0,62
	####	0	9,3E-05	3,21	2	#######	-0,07
	####	0	0,00142	3,20	3	#######	0,25

	####	0	0,00103	3,20	2	#######	-0,37
	####	0	0,00068	3,20	2	#######	0,86
	####	0	0,00015	3,18	2	#######	-0,45
M5(Oxidation)	####	0	0,00052	3,18	2	#######	1,40
	####	0	0,00051	3,18	3	#######	-0,60
	####	0	0,0038	3,18	2	#######	-0,45
	####	0	0,00451	3,17	2	#######	0,04
	####	0	0,00211	3,17	2	#######	0,00
	####	0	4,4E-05	3,16	3	#######	-1,72
	####	0	0,00586	3,16	3	#######	4,92
	####	0	0,00272	3,15	2	#######	-0,30
C9(Carbamidomethyl)	####	0	0,00202	3,14	2	#######	0,05
	####	0	0,00243	3,12	2	#######	1,67
M5(Oxidation)	####	0	0,00474	3,12	2	#######	-1,21
	####	0	0,00111	3,12	2	#######	-0,80
	####	0	8,6E-05	3,10	2	#######	-0,02
	####	0	4,7E-06	3,10	2	#######	-0,72
	####	0	0,00163	3,09	2	938,52941	-1,28
	####	0	0,00038	3,08	2	#######	-0,70
	####	0	0,00022	3,08	2	#######	-0,02
	####	0	0,00028	3,08	2	991,59300	-0,54
	####	0	0,00216	3,07	2	920,48241	-1,34
	####	0	0,0018	3,07	2	#######	-0,49
	####	0	0,00152	3,07	2	#######	-0,59
	####	0	0,00141	3,07	2	#######	-1,92
M4(Oxidation)	####	0	0,00287	3,06	2	997,44023	-0,59
	####	0	0,00123	3,05	2	#######	-0,76
	####	0	0,00027	3,05	2	#######	-0,30
	####	0	0,00081	3,03	3	#######	-1,60
	####	0	0,00063	3,01	2	#######	-0,25
	####	0	0,00058	3,00	3	#######	1,96
M3(Oxidation); M4(Oxidation)	####	0	0,00325	3,00	2	#######	-0,83
	####	0	0,00025	2,98	2	#######	-1,26
C6(Carbamidomethyl)	####	0	0,00036	2,98	3	#######	0,68
M2(Oxidation); M4(Oxidation); M5(Oxidation)	####	0	8,6E-05	2,98	2	#######	-0,07
	####	0	0,00235	2,97	2	947,42064	-1,21
	####	0	0,00215	2,97	2	#######	-1,03
	####	0	0,00425	2,96	2	934,50212	-1,29
M11(Oxidation)	####	0	3,8E-05	2,96	3	#######	-0,20
	####	0	0,00384	2,96	2	#######	-1,79
	####	0	0,00179	2,95	2	#######	0,27
	####	0	0,00014	2,95	2	#######	-0,30
	####	0	0,00428	2,95	2	861,47936	0,53
	####	0	5,4E-05	2,95	2	#######	-0,95
	####	0	0,00089	2,95	2	987,48955	0,12
	####	0	6,5E-05	2,95	2	968,61382	-0,14
M3(Oxidation)	####	0	0,00088	2,93	2	963,48174	0,13
C2(Carbamidomethyl)	####	0	0,0008	2,93	3	#######	-1,32
	####	0	0,00249	2,92	2	955,44780	-0,18
	####	0	0,00103	2,92	2	996,51097	0,05
	####	0	0,00244	2,92	2	#######	0,10
	####	0	0,00136	2,91	2	#######	-1,01
	####	0	2,4E-05	2,91	3	#######	0,20
	####	0	0,00022	2,91	3	#######	-0,33
	####	0	0,00231	2,90	2	826,51396	-0,72
	####	0	0,00095	2,89	3	#######	-0,38
	####	0	0,00088	2,88	2	#######	-0,28
	####	0	0,00573	2,88	2	988,46257	0,34
	####	0	0,00079	2,88	2	#######	-1,42

C14(Carbamidomethyl); C16(Carbamidomethyl); M17(Oxidation)	####	0	0,00063	2,87	2	#######	0,74
M4(Oxidation)	####	0	0,00215	2,87	3	#######	0,53
	####	0	0,00105	2,86	2	#######	-1,66
	####	0	0,00027	2,86	3	#######	0,70
	####	0	0,0014	2,86	2	887,51952	-0,22
	####	0	0,00114	2,85	2	975,56182	-0,44
	####	0	0,00233	2,84	2	947,51598	0,32
	####	0	0,00594	2,84	2	#######	0,12
	####	0	0,00039	2,84	3	#######	-0,09
	####	0	0,00087	2,82	2	#######	0,06
	####	0	0,00065	2,82	4	#######	0,43
	####	0	1,3E-05	2,81	2	#######	0,45
	####	0	0,006	2,81	2	#######	0,28
	####	0	0,00217	2,80	2	948,52696	0,84
	####	0	0,00588	2,80	3	#######	-0,43
	####	0	0,00249	2,78	2	944,47215	-0,26
M8(Oxidation)	####	0	0,00271	2,77	2	#######	-0,64
M16(Oxidation)	####	0	0,00541	2,76	3	#######	-1,33
	####	0	5,7E-05	2,75	3	#######	-0,60
	####	0	0,00053	2,74	2	921,51476	-0,58
M3(Oxidation)	####	0	0,00365	2,74	2	#######	-0,90
	####	0	0,00041	2,73	3	#######	-0,06
	####	0	0,00257	2,72	2	#######	-0,25
	####	0	2,8E-05	2,70	3	#######	-0,50
	####	0	0,00105	2,69	2	982,49370	-1,59
	####	0	0,00214	2,63	3	#######	1,05
	####	0	6,3E-05	2,63	3	#######	-1,45
	####	0	0,00482	2,62	2	850,52471	-1,26
	####	0	0,00153	2,62	2	#######	0,85
	####	0	0,00066	2,61	2	#######	-0,71
M5(Oxidation)	####	0	0,00275	2,60	2	#######	-0,18
	####	0	0,0002	2,60	3	#######	-0,67
	####	0	0,00356	2,59	2	889,50987	-0,37
	####	0	0,00117	2,59	2	891,45342	0,38
	####	0	0,00339	2,57	2	965,48381	-0,18
	####	0	0,00287	2,54	2	#######	0,12
	####	0	0,00023	2,53	4	#######	1,48
	####	0	0,00228	2,51	2	#######	-0,47
	####	0	0,0009	2,49	2	#######	-0,23
C5(Carbamidomethyl); C8(Carbamidomethyl)	####	0	0,00031	2,49	3	#######	0,03
	####	0	0,00335	2,42	2	858,50395	-0,51
	####	0	0,00542	2,35	2	954,45305	0,32
	####	0	0,00165	2,31	2	#######	-0,65
	####	0	0,00348	2,28	4	#######	0,48
	####	0	0,00346	2,27	3	#######	-0,18
	####	0	0,00131	2,23	4	#######	1,60
	####	0	0,00426	2,18	2	875,53484	-0,14
	####	0	4,5E-05	2,15	4	#######	-0,02
	####	0	0,00531	2,07	3	#######	-2,38
	####	0	7E-05	1,89	3	#######	-0,29
	####	0	0,00042	1,86	4	#######	-0,46
	####	0	1,9E-05	1,85	4	#######	-0,04
M8(Oxidation)	####	0,001	0,07841	4,31	3	#######	0,28
	####	0,001	0,05677	4,28	3	#######	-4,12
C31(Carbamidomethyl)	####	0,001	0,02218	4,15	3	#######	-5,19
	####	0,001	0,03788	4,09	3	#######	0,27
	####	0,001	0,05025	4,05	3	#######	0,97
	####	0,001	0,0107	4,04	3	#######	-0,79
	####	0,001	0,00679	3,92	3	#######	1,13

	####	0,001	0,01002	3,89	3	#######	-2,57
C7(Carbamidomethyl)	####	0,001	0,00847	3,77	2	#######	-0,32
	####	0,001	0,00743	3,68	2	#######	-0,50
	####	0,001	0,01053	3,64	2	#######	1,09
M10(Oxidation)	####	0,001	0,01526	3,58	3	#######	-0,42
C5(Carbamidomethyl); C10(Carbamidomethyl)	####	0,001	0,01441	3,58	2	#######	-0,65
	####	0,001	0,00949	3,56	2	#######	-0,27
	####	0,001	0,03732	3,56	2	#######	5,43
	####	0,001	0,00767	3,56	2	#######	-0,69
M11(Oxidation)	####	0,001	0,07928	3,53	3	#######	0,15
M3(Oxidation)	####	0,001	0,00796	3,52	3	#######	1,03
	####	0,001	0,01911	3,51	2	#######	-2,36
	####	0,001	0,00601	3,50	4	#######	3,24
	####	0,001	0,02481	3,49	2	#######	-0,71
	####	0,001	0,00677	3,46	2	#######	-0,63
	####	0,001	0,009	3,44	2	#######	0,35
	####	0,001	0,00881	3,42	2	#######	0,18
	####	0,001	0,017	3,41	2	#######	-0,67
	####	0,001	0,058	3,41	2	#######	0,70
M3(Oxidation)	####	0,001	0,00613	3,40	3	#######	-0,41
M5(Oxidation)	####	0,001	0,06986	3,40	3	#######	1,80
	####	0,001	0,01654	3,38	3	#######	0,15
	####	0,001	0,0747	3,38	2	#######	2,70
	####	0,001	0,06521	3,37	2	929,52910	-1,26
	####	0,001	0,00623	3,34	2	#######	-0,94
	####	0,001	0,0647	3,34	2	#######	-1,73
C21(Carbamidomethyl); C29(Carbamidomethyl)	####	0,001	0,0147	3,31	4	#######	-2,70
M1(Oxidation); M5(Oxidation)	####	0,001	0,01305	3,30	2	#######	2,19
M7(Oxidation)	####	0,001	0,0089	3,30	2	#######	-1,69
	####	0,001	0,00834	3,29	4	#######	-2,75
M8(Oxidation)	####	0,001	0,05942	3,26	2	#######	-0,17
C4(Carbamidomethyl)	####	0,001	0,02478	3,24	3	#######	0,17
	####	0,001	0,0277	3,21	2	#######	-0,35
X8(L)	####	0,001	0,01625	3,17	2	#######	-0,90
	####	0,001	0,00604	3,17	2	#######	1,15
M10(Oxidation)	####	0,001	0,02749	3,17	2	#######	-3,03
M14(Oxidation)	####	0,001	0,01074	3,15	3	#######	-0,80
	####	0,001	0,01033	3,11	3	#######	-0,06
	####	0,001	0,01175	3,10	2	#######	-0,24
	####	0,001	0,01144	3,10	2	851,42595	0,19
	####	0,001	0,03057	3,10	2	#######	-0,71
	####	0,001	0,02501	3,09	2	#######	-0,23
	####	0,001	0,00618	3,07	2	#######	0,13
	####	0,001	0,0286	3,04	2	958,51097	0,46
	####	0,001	0,01126	3,03	2	#######	-2,27
	####	0,001	0,00612	3,03	2	#######	-0,75
	####	0,001	0,01312	3,03	2	#######	-0,07
M8(Oxidation)	####	0,001	0,03323	3,02	2	#######	-0,32
M3(Oxidation)	####	0,001	0,00821	3,02	2	#######	-1,52
	####	0,001	0,01138	3,01	2	#######	3,86
C6(Carbamidomethyl)	####	0,001	0,01093	2,99	2	#######	0,10
	####	0,001	0,00725	2,98	3	#######	-0,77
	####	0,001	0,0331	2,97	3	#######	0,63
	####	0,001	0,01624	2,96	2	#######	-1,23
	####	0,001	0,01499	2,92	3	#######	0,93
	####	0,001	0,02396	2,92	2	#######	-1,00
	####	0,001	0,00695	2,92	2	#######	0,27
M4(Oxidation)	####	0,001	0,00716	2,91	3	#######	-1,46
	####	0,001	0,00987	2,91	2	#######	-0,33

	####	0,001	0,0864	2,89	2	#######	5,54
	####	0,001	0,01452	2,89	2	901,47362	-0,22
C7(Carbamidomethyl); C14(Carbamidomethyl)	####	0,001	0,02101	2,88	2	#######	2,33
	####	0,001	0,05586	2,88	2	#######	-0,37
	####	0,001	0,00711	2,88	2	976,45232	-0,03
	####	0,001	0,01804	2,86	3	#######	0,22
	####	0,001	0,02516	2,86	2	#######	-1,10
	####	0,001	0,02592	2,86	4	#######	-1,06
	####	0,001	0,01018	2,86	2	#######	-1,71
	####	0,001	0,02842	2,86	2	910,42546	-1,17
	####	0,001	0,0065	2,86	2	#######	-0,54
	####	0,001	0,00972	2,82	2	871,46337	0,13
	####	0,001	0,02858	2,80	2	934,49913	-0,19
	####	0,001	0,00691	2,80	2	#######	-1,48
	####	0,001	0,0249	2,79	2	#######	0,62
	####	0,001	0,04789	2,77	2	906,54082	0,05
	####	0,001	0,0216	2,77	2	#######	0,16
	####	0,001	0,03623	2,76	2	#######	-0,99
	####	0,001	0,072	2,74	2	816,45647	-1,18
M18(Oxidation); M23(Oxidation)	####	0,001	0,07146	2,73	5	#######	2,13
	####	0,001	0,04133	2,73	2	970,62981	0,21
M6(Oxidation)	####	0,001	0,0837	2,73	2	#######	-0,54
	####	0,001	0,06833	2,72	2	#######	-0,89
C8(Carbamidomethyl)	####	0,001	0,054	2,72	2	#######	-1,60
	####	0,001	0,0813	2,72	2	799,50237	-1,61
	####	0,001	0,02199	2,72	2	#######	-0,11
	####	0,001	0,00637	2,70	3	#######	-2,42
M4(Oxidation)	####	0,001	0,01088	2,69	2	835,39781	-0,09
	####	0,001	0,00943	2,68	2	927,46410	-0,24
	####	0,001	0,02269	2,68	2	#######	-0,03
	####	0,001	0,00887	2,68	2	#######	-0,31
	####	0,001	0,05579	2,68	3	#######	-2,70
	####	0,001	0,04344	2,67	2	#######	-0,22
	####	0,001	0,0189	2,66	2	939,55095	-0,06
	####	0,001	0,02939	2,66	2	917,53081	0,59
	####	0,001	0,03725	2,64	2	865,40520	0,16
	####	0,001	0,01315	2,64	2	#######	-0,73
	####	0,001	0,01053	2,63	2	#######	1,50
	####	0,001	0,01395	2,63	2	#######	-0,67
	####	0,001	0,01742	2,61	3	#######	3,71
	####	0,001	0,01029	2,60	2	875,49443	-0,14
	####	0,001	0,01729	2,59	2	964,44573	-2,70
	####	0,001	0,03934	2,59	2	#######	-2,57
C5(Carbamidomethyl)	####	0,001	0,03973	2,59	2	#######	0,30
	####	0,001	0,05245	2,58	2	812,49822	-0,86
M4(Oxidation)	####	0,001	0,01043	2,58	2	#######	0,26
	####	0,001	0,01723	2,57	2	871,50041	0,90
	####	0,001	0,08023	2,55	2	#######	0,83
	####	0,001	0,01949	2,51	2	824,42455	-1,92
	####	0,001	0,05318	2,50	3	#######	-0,97
	####	0,001	0,02138	2,50	3	#######	-0,84
	####	0,001	0,03948	2,49	2	#######	-0,42
	####	0,001	0,0239	2,48	2	932,47905	-0,61
M5(Oxidation)	####	0,001	0,03674	2,47	3	#######	-1,18
	####	0,001	0,04236	2,46	2	883,44652	0,43
	####	0,001	0,0206	2,46	2	#######	-0,37
	####	0,001	0,01927	2,45	3	#######	-0,19
	####	0,001	0,07533	2,45	2	#######	0,96
	####	0,001	0,02093	2,44	2	#######	-0,06

	####	0,001	0,01091	2,43	4	#######	0,63
	####	0,001	0,0303	2,43	2	#######	-2,37
	####	0,001	0,05184	2,43	2	833,41472	-0,61
	####	0,001	0,00812	2,43	2	828,49352	-0,37
	####	0,001	0,03489	2,41	2	#######	-1,56
	####	0,001	0,04178	2,39	2	896,49529	0,45
	####	0,001	0,01135	2,39	2	819,40105	-2,35
	####	0,001	0,01462	2,38	2	877,47728	-0,64
	####	0,001	0,07336	2,37	2	#######	-1,87
	####	0,001	0,02296	2,37	2	882,51561	0,00
	####	0,001	0,01881	2,36	2	#######	0,14
	####	0,001	0,04103	2,36	2	#######	0,97
M3(Oxidation)	####	0,001	0,00721	2,34	3	#######	0,21
	####	0,001	0,03632	2,34	2	962,59172	-0,42
C3(Carbamidomethyl)	####	0,001	0,02016	2,34	3	#######	0,64
	####	0,001	0,03446	2,34	2	#######	-0,38
	####	0,001	0,01154	2,34	2	#######	-0,76
M5(Oxidation)	####	0,001	0,0662	2,33	3	#######	0,87
	####	0,001	0,03278	2,32	2	857,45152	-0,09
C8(Carbamidomethyl)	####	0,001	0,05282	2,30	2	912,42473	0,35
	####	0,001	0,06706	2,30	2	929,53075	0,51
	####	0,001	0,01115	2,30	2	867,45683	-0,28
	####	0,001	0,06525	2,29	2	824,45213	1,05
	####	0,001	0,04558	2,29	2	836,43065	0,60
	####	0,001	0,07936	2,28	3	#######	-0,85
C2(Carbamidomethyl)	####	0,001	0,03002	2,27	2	868,39665	-1,79
M7(Oxidation)	####	0,001	0,00737	2,25	2	834,47557	0,20
	####	0,001	0,02143	2,24	2	989,58745	-0,33
M2(Oxidation)	####	0,001	0,08084	2,23	2	875,43273	-0,53
	####	0,001	0,01637	2,17	3	#######	0,12
	####	0,001	0,04921	2,15	2	887,49455	-0,01
C4(Carbamidomethyl)	####	0,001	0,0793	2,15	2	828,40288	-0,49
	####	0,001	0,01348	2,13	2	835,49193	-0,59
	####	0,001	0,0362	2,13	2	858,50420	-0,21
M2(Oxidation)	####	0,001	0,06397	2,11	2	879,39794	-1,15
	####	0,001	0,07963	2,08	2	#######	-2,33
C6(Carbamidomethyl)	####	0,001	0,08383	2,07	2	946,43401	0,11
	####	0,001	0,03053	2,06	2	#######	-0,91
	####	0,001	0,00824	2,04	3	#######	-0,77
M4(Oxidation)	####	0,001	0,06778	2,04	3	#######	-0,30
C2(Carbamidomethyl)	####	0,001	0,07841	2,04	2	911,40465	0,71
	####	0,001	0,06286	2,03	2	#######	-0,47
	####	0,001	0,00612	1,99	2	#######	-1,61
	####	0,001	0,02476	1,97	2	824,41478	-0,13
	####	0,001	0,02241	1,93	2	902,44768	-0,28
	####	0,001	0,07029	1,91	2	821,47563	-1,37
	####	0,001	0,01131	1,90	2	961,51000	-0,21
M5(Oxidation)	####	0,001	0,07125	1,88	2	879,41026	-2,96
	####	0,001	0,05187	1,86	2	#######	-0,28
	####	0,001	0,0164	1,86	2	859,43828	0,01
	####	0,001	0,06383	1,85	3	#######	0,63
	####	0,001	0,08758	1,80	2	887,42577	-0,02
	####	0,001	0,02414	1,75	2	912,49364	-0,18
	####	0,001	0,07713	1,74	3	#######	1,63
	####	0,001	0,00845	1,73	3	#######	0,18
	####	0,001	0,05158	1,72	3	#######	-0,84
	####	0,001	0,06445	1,71	3	#######	-1,22
M2(Oxidation)	####	0,001	0,08659	1,64	2	#######	-1,03
	####	0,001	0,07044	1,30	4	#######	-1,17

	####	0,001	0,0071	1,26	3	#######	0,23
	####	0,002	0,09464	2,80	2	948,47386	-0,73
M7(Oxidation)	####	0,002	0,09095	2,78	2	#######	0,21
M4(Oxidation)	####	0,002	0,09467	2,52	3	#######	-0,45
M3(Oxidation)	####	0,002	0,09151	2,36	2	864,42418	-0,30
	####	0,002	0,09547	2,33	2	931,58202	-0,30
C1(Carbamidomethyl)	####	0,002	0,09561	2,07	2	856,38671	-0,29
	####	0,002	0,09583	1,63	2	822,38939	0,10
	####	0,002	0,08821	1,60	2	985,59319	0,32
	####	0,002	0,09455	0,95	3	#######	-2,25
M22(Oxidation)	####	0,003	0,1363	3,27	3	#######	-0,59
	####	0,003	0,1368	3,06	2	#######	-1,82
	####	0,003	0,1043	2,93	2	#######	-0,58
	####	0,003	0,1015	2,77	2	973,50658	0,41
	####	0,003	0,1165	2,64	2	#######	-2,28
	####	0,003	0,1424	2,64	3	#######	-0,38
	####	0,003	0,1222	2,57	2	999,55815	-0,06
C9(Carbamidomethyl); M10(Oxidation)	####	0,003	0,1239	2,55	3	#######	-1,37
	####	0,003	0,142	2,53	2	#######	-0,01
M6(Oxidation)	####	0,003	0,1048	2,45	2	#######	-1,24
	####	0,003	0,1183	2,33	2	988,46068	-1,57
	####	0,003	0,1363	2,30	2	991,50511	-0,39
	####	0,003	0,1137	2,30	2	870,50359	-0,92
M1(Oxidation)	####	0,003	0,1074	2,29	2	963,49437	1,57
	####	0,003	0,1227	2,21	2	929,53075	0,51
	####	0,003	0,1313	2,17	3	#######	-0,09
	####	0,003	0,1234	2,10	2	906,49297	-0,20
	####	0,003	0,1325	2,09	3	#######	-0,60
M3(Oxidation)	####	0,004	0,1566	2,95	2	#######	-0,26
	####	0,004	0,1545	2,81	2	#######	-0,04
	####	0,004	0,1608	2,81	2	#######	-1,28
	####	0,004	0,1442	2,62	2	#######	-0,41
	####	0,004	0,1482	2,42	3	#######	0,30
	####	0,004	0,1598	2,41	2	#######	-0,66
	####	0,004	0,1558	2,29	2	#######	0,90
	####	0,004	0,1556	2,27	3	#######	1,06
M11(Oxidation); M13(Oxidation)	####	0,004	0,1581	2,17	3	#######	-1,18
	####	0,004	0,1611	2,09	2	960,47667	-1,97
	####	0,004	0,1485	2,04	2	862,44115	-0,73
C20(Carbamidomethyl)	####	0,005	0,1643	2,34	2	#######	0,20
	####	0,005	0,1654	2,19	2	875,52983	-1,25
	####	0,006	0,1763	3,24	2	#######	0,70
	####	0,006	0,1755	2,83	2	#######	0,31
	####	0,006	0,1791	2,70	2	#######	-1,43
	####	0,006	0,1746	2,65	2	807,43505	-1,15
	####	0,006	0,1743	2,23	2	#######	-0,04
	####	0,006	0,1939	2,10	2	816,48216	-0,52
M16(Oxidation); C22(Carbamidomethyl)	####	0,006	0,1822	1,25	4	#######	1,56
M15(Oxidation)	####	0,007	0,1993	3,24	3	#######	-6,53
	####	0,007	0,212	2,97	2	#######	-2,27
	####	0,007	0,2115	2,68	2	997,51018	0,00
	####	0,007	0,2072	2,38	2	#######	0,64
	####	0,007	0,2136	2,31	2	#######	0,67
	####	0,007	0,1987	2,07	4	#######	0,37
	####	0,007	0,2171	1,99	3	#######	-1,23
M2(Oxidation)	####	0,007	0,2017	1,91	2	823,41240	-0,89
	####	0,007	0,2078	1,30	3	#######	-1,15
M5(Oxidation)	####	0,008	0,2252	2,82	3	#######	-0,25
	####	0,008	0,2267	2,71	2	911,42278	1,09

	####	0,008	0,2282	2,60	2	#######	1,35
	####	0,008	0,2238	2,06	2	#######	-0,70
	####	0,008	0,2299	2,00	2	843,42497	5,04
	####	0,009	0,2474	3,18	3	#######	-0,66
	####	0,009	0,2352	2,95	2	#######	1,22
	####	0,009	0,2456	2,49	2	980,52635	-0,94
	####	0,009	0,2375	2,46	3	#######	-0,03
	####	0,009	0,2429	2,09	2	948,48937	-0,45
	####	0,009	0,2557	1,79	2	801,39897	-1,58
C7(Carbamidomethyl)	####	0,009	0,2557	0,24	2	#######	-5,47

RT [min]	# Missed Cleavages
62,36	0
65,58	0
56,57	0
50,84	1
63,77	0
74,71	0
70,27	0
57,44	0
71,38	0
83,14	0
75,34	0
71,83	0
61,94	1
65,12	0
47,87	0
77,12	0
67,66	0
59,34	0
64,54	0
71,30	0
65,63	0
63,09	0
65,05	0
59,46	0
63,94	0
64,48	0
56,81	0
67,27	1
65,49	0
63,50	0
79,10	0
49,45	0
63,12	0
47,32	0
59,19	1
71,36	0
64,36	0
60,24	0
60,16	0
63,09	0
81,31	0
82,81	0
60,89	0
40,74	0
69,38	0
67,66	0
71,87	0
71,28	0
55,57	0
62,80	0
52,17	0
69,54	0
61,82	0
45,04	0
48,78	0

78,45	0
67,59	0
52,29	0
63,84	0
57,60	0
59,52	1
57,21	0
59,47	0
64,66	0
70,34	0
65,42	0
52,85	0
64,03	0
58,74	0
57,60	1
76,46	0
65,31	0
59,12	0
68,44	0
63,42	0
57,40	1
56,81	0
63,58	0
55,08	0
66,28	0
38,41	0
62,32	0
68,21	0
47,88	1
76,58	1
52,20	0
65,64	0
78,25	0
66,24	0
58,07	0
61,83	0
51,73	0
61,59	0
46,69	0
75,34	0
55,67	0
70,83	0
75,88	0
48,44	1
52,03	0
45,70	0
57,33	0
52,41	0
52,27	1
76,97	0
71,51	0
61,79	0
53,17	0
58,27	0
50,37	0
75,86	0
70,22	0
60,87	0
58,08	0

65,02	0
55,27	0
49,21	0
38,20	0
69,90	0
69,23	1
64,20	1
56,99	1
51,54	0
46,99	0
34,60	0
55,03	0
60,55	0
70,44	0
24,00	1
70,85	0
53,13	0
61,25	0
63,25	0
62,34	0
63,92	0
48,47	0
62,78	0
54,35	1
71,03	0
46,54	0
73,62	0
69,05	0
58,14	0
67,68	0
51,58	0
74,10	0
71,81	0
71,01	0
61,36	0
59,50	0
63,68	0
58,37	0
55,18	0
52,35	0
67,72	0
69,78	0
51,33	0
69,32	0
70,85	0
60,36	0
52,43	1
50,95	1
85,60	0
65,91	0
38,96	1
47,61	0
54,14	0
87,10	0
72,13	0
49,76	0
62,24	0
75,57	0
72,02	0

67,34	0
77,89	0
65,24	0
65,97	1
41,65	0
71,17	0
49,95	2
54,71	0
70,49	0
63,27	0
57,91	0
57,37	0
61,26	0
56,14	0
38,85	1
59,48	0
70,65	0
59,66	0
61,53	0
60,95	0
64,65	0
73,77	0
45,16	0
75,80	0
51,46	0
86,16	0
66,24	0
67,42	0
51,68	1
66,96	0
70,64	0
75,88	0
65,21	0
83,87	0
64,13	0
70,00	0
47,13	1
75,40	0
57,96	0
61,94	0
51,10	0
52,20	0
60,24	0
65,65	0
74,10	0
61,68	0
44,65	0
85,52	0
57,73	0
64,85	0
46,58	0
55,69	0
56,47	0
68,75	0
56,87	0
30,10	0
61,06	0
58,79	0
55,22	0

52,53	0
37,56	1
69,17	0
55,05	1
63,68	1
73,47	0
83,04	0
69,19	0
44,76	0
54,08	0
62,21	0
53,50	0
51,09	0
43,66	0
46,25	1
74,27	0
58,66	0
71,82	0
63,64	0
53,13	0
57,03	0
40,37	1
62,61	0
28,54	0
64,99	0
63,58	0
48,78	0
68,88	1
69,65	0
61,44	0
54,68	0
72,58	0
65,53	0
65,04	0
68,33	0
61,30	1
47,81	0
69,66	0
80,98	0
76,37	0
43,86	0
54,64	0
53,14	0
77,56	0
80,68	0
67,14	0
68,64	0
69,01	0
63,72	0
67,39	0
53,10	0
55,03	0
74,82	0
59,41	0
56,04	0
59,43	0
67,18	0
68,79	0
76,22	0

48,04	0
58,93	1
57,29	0
56,57	0
52,96	0
58,67	0
61,07	0
60,31	0
54,39	1
48,98	0
35,54	0
71,52	1
62,84	1
49,39	0
64,74	1
55,57	0
59,53	0
71,29	0
46,01	0
51,27	0
52,24	0
60,57	0
46,38	0
85,03	0
55,05	0
41,37	0
81,00	0
62,16	0
40,97	0
60,16	0
65,47	0
35,44	0
70,45	0
74,77	0
54,75	0
31,69	0
42,01	0
41,99	0
42,34	0
77,14	0
47,99	0
42,62	0
81,08	0
80,89	0
57,81	0
67,19	0
64,96	0
48,43	0
69,72	0
70,17	0
42,50	0
65,63	0
50,17	1
65,20	0
40,27	0
48,12	0
59,79	0
76,85	0
72,15	0

51,86	1
45,79	0
42,21	0
63,50	0
64,77	0
45,96	0
61,24	0
53,91	0
34,51	0
52,37	0
44,80	0
44,21	0
52,92	0
76,71	0
37,70	0
71,85	1
33,49	0
46,57	0
43,94	0
62,00	0
35,78	0
48,50	0
55,56	0
57,39	0
44,64	0
70,80	0
52,21	0
67,88	0
68,58	0
66,00	0
72,77	0
41,06	1
89,23	0
51,71	1
63,77	1
61,47	0
60,30	0
67,70	0
59,47	0
58,49	0
64,09	0
71,84	0
46,56	0
51,51	0
82,18	0
36,10	0
50,73	0
30,27	0
30,20	0
65,95	0
49,00	0
76,63	0
72,64	0
65,47	0
47,04	0
52,82	0
68,62	0
62,72	0
43,17	0

43,65	1
51,92	0
36,73	0
59,69	0
61,05	0
59,63	0
39,59	0
65,70	0
46,62	0
44,16	0
47,25	0
57,40	1
73,32	0
49,59	0
81,03	1
74,58	0
46,49	0
40,24	0
45,95	0
58,44	1
58,56	0
52,59	0
63,62	0
57,00	0
29,95	0
60,01	0
44,92	0
57,96	0
29,56	1
46,37	0
81,80	0
44,14	0
59,75	0
37,46	0
71,62	0
82,38	0
27,75	0
48,36	0
70,13	0
37,84	0
35,74	0
41,18	0
81,16	0
56,87	0
65,46	0
40,27	0
73,33	0
41,68	0
53,50	0
60,97	0
69,04	0
71,21	0
46,97	0
33,91	0
28,42	0
52,57	0
52,26	1
60,16	0
57,53	1

66,55	0
39,80	0
33,43	1
39,62	0
46,77	0
56,73	0
57,68	0
57,57	0
71,96	0
40,92	0
81,21	0
50,74	0
57,94	0
55,53	0
61,00	1
56,19	0
39,37	0
47,72	0
66,32	0
41,61	0
56,43	0
50,44	0
62,72	0
47,02	0
78,36	0
75,52	0
70,94	0
38,30	0
55,19	0
45,92	0
63,32	0
77,13	0
55,23	0
51,59	0
49,89	0
48,81	0
63,46	0
38,89	0
55,44	0
35,38	0
61,35	1
43,59	0
53,35	1
54,96	0
33,37	0
54,89	1
41,76	0
80,40	0
53,36	0
45,50	0
80,79	0
26,10	0
41,82	0
40,02	0
55,89	0
46,63	0
61,00	0
55,78	1
51,05	0

50,14	0
39,23	0
38,31	0
38,62	0
25,62	0
45,30	0
71,96	0
59,82	0
47,49	0
43,45	0
74,83	0
55,63	0
28,46	0
75,09	0
55,00	0
42,97	0
44,63	0
69,89	0
50,07	0
40,19	1
38,57	0
77,65	0
30,55	0
44,84	0
39,86	0
49,18	0
49,51	0
25,88	0
45,71	0
27,99	0
42,36	0
82,69	0
68,69	0
72,72	0
57,34	0
58,18	0
56,88	0
59,55	0
50,45	0
42,43	0
58,78	0
49,91	0
48,10	0
38,34	0
76,54	0
47,61	0
65,71	0
48,16	1
44,87	0
41,06	0
61,54	0
50,97	0
44,59	0
42,25	0
49,16	0
46,07	0
46,36	0
26,37	0
53,36	1

45,48	0
30,51	0
40,86	0
53,20	0
55,13	0
54,97	0
40,77	0
58,42	0
65,08	0
40,34	0
28,05	0
78,07	0
37,42	0
47,78	0
37,55	0
39,46	0
35,97	0
46,88	0
71,23	0
55,11	0
65,77	0
72,55	0
52,58	0
49,99	0
72,97	0
53,35	0
42,67	0
59,78	0
28,11	0
60,08	0
74,43	0
73,07	0
49,22	0
39,71	0
38,84	0
53,04	0
60,78	0
45,08	0
61,83	0
63,63	0
81,26	0
52,16	0
54,85	1
59,13	0
51,95	0
34,79	0
59,61	0
53,32	0
30,63	0
70,30	0
55,25	0
54,63	0
30,32	0
48,39	0
29,76	0
71,35	0
54,96	0
48,06	0
63,39	0

53,39	0
32,52	0
46,19	0
50,38	0
61,60	0
47,49	0
58,89	0
49,48	0
33,73	0
33,38	0
38,61	0
46,97	0
44,44	0
61,02	0
47,21	0
47,99	0
61,52	0
58,25	0
35,13	1
48,83	0
49,96	0
39,74	0
58,06	0
49,44	0
64,00	1
33,88	0
53,73	0
50,66	2
60,56	0
42,43	0
45,74	0
65,59	0
45,54	1
37,29	1
57,30	0
39,53	1
37,35	0
47,62	0
55,63	1
57,10	0
40,04	0
62,85	0
74,18	0
61,55	0
18,66	0
68,85	0
60,53	0
19,12	0
42,96	0
56,82	0
48,50	0
61,25	0
43,60	1
51,31	0
63,07	0
56,11	0
50,60	0
48,80	0
45,67	0

52,69	0
34,99	1
70,63	0
58,43	0
62,50	0
30,41	0
51,92	0
61,68	0
46,64	0
54,62	0
49,83	0
70,52	0
29,83	0
44,44	0
37,60	0
57,63	0
82,62	0
50,12	0
30,33	0
44,32	0
58,94	0
59,91	1
42,30	0
40,69	0
40,01	0
59,33	0
41,55	0
24,50	0
47,05	0
29,85	0
68,69	0
43,11	0
45,35	0
85,20	0
40,99	0
54,11	0
40,75	0
38,24	0
51,18	0
33,05	0
59,08	0
30,79	0
44,62	0
42,80	0
40,74	0
82,42	0
57,22	0
46,91	0
57,27	0
61,55	0
30,19	0
33,54	0
60,09	0
52,98	0
44,21	0
59,26	0
47,57	0
41,47	0
46,08	0

57,29	0
47,28	0
40,16	0
35,32	0
49,92	0
30,15	0
37,04	0
48,62	0
34,75	0
84,23	0
23,70	0
33,89	0
61,92	0
46,43	0
42,19	0
59,05	0
52,34	0
40,13	0
34,91	0
65,09	0
49,94	0
44,06	0
64,92	0
56,57	0
66,88	0
32,95	0
44,86	0
69,82	0
79,20	0
57,66	0
39,36	0
44,58	0
47,17	0
39,77	0
27,14	0
43,57	0
44,72	0
51,31	0
46,79	0
25,99	0
38,38	0
71,44	0
24,05	0
57,66	0
30,51	0
48,09	0
30,27	0
52,28	0
26,15	0
17,35	0
27,28	0
64,09	0
54,06	0
45,02	0
43,81	0
71,91	1
54,74	0
43,67	0
28,24	0

41,93	0
41,29	1
45,66	0
45,84	0
42,54	0
57,84	0
25,17	0
46,91	0
61,33	1
47,02	0
45,68	0
52,28	0
44,95	0
39,66	0
65,94	0
45,95	0
35,37	0
50,62	1
53,16	0
44,87	0
47,89	0
48,15	0
30,21	0
55,79	0
24,64	0
39,96	1
56,72	0
35,04	0
37,48	0
86,26	0
49,34	0
59,38	0
30,54	0
24,46	0
45,04	0
50,77	0
66,60	0
48,91	0
51,94	0
57,35	0
43,37	0
22,32	0
34,84	0
41,90	0
24,36	0
67,57	1
62,25	0
37,38	0
40,06	0
51,13	0
51,27	1
55,09	0
59,44	2
55,96	0
63,98	0
32,11	1
39,98	1
56,31	0
54,04	0

82,64	0
52,20	0
40,86	0
61,60	0
42,33	1
35,55	0
52,72	0
55,39	0
34,45	1
34,61	0
83,02	1
28,92	0
73,45	0
39,42	0
26,03	0
76,89	0
51,70	0
48,74	0
64,25	0
17,03	1
44,99	0
56,76	0
41,44	0
38,33	0
33,80	0
41,50	0
59,03	1
42,98	0
32,94	0
66,34	1
43,47	0
34,53	1
45,09	1
88,56	0
38,76	0
29,54	0
46,89	0
41,21	1
31,75	1
27,96	0
79,86	0
45,08	0
53,45	0
35,83	0
40,70	0
62,51	0
46,29	0
56,73	0
45,31	0
49,57	0
48,10	0
22,30	1
32,35	1
39,15	0
48,99	1
48,75	0
46,12	0
33,45	1
20,03	1

43,29	0
31,66	0
56,79	0
26,92	0
59,59	0
15,67	1
23,54	0
43,90	2
40,41	0
39,28	0
46,80	0
29,87	0
43,58	0
37,52	0
67,73	0
36,28	0
41,88	0
43,50	0
30,65	0
58,69	0
48,24	0
20,43	0
48,16	0
30,47	0
28,77	0
39,98	0
72,00	0
24,30	0
20,61	0
30,28	0
58,85	0
42,50	0
47,96	0
43,17	0
40,71	0
23,90	0
44,28	0
55,98	0
46,64	0
44,10	0
26,91	0
23,62	0
37,94	0
46,82	0
45,40	0
22,47	0
30,89	0
60,96	0
27,12	0
34,76	1
46,11	1
57,56	0
24,66	0
71,70	0
53,90	0
39,60	0
71,00	1
60,60	0
18,28	0

51,78	0
42,53	0
30,73	0
35,72	0
37,94	0
24,36	0
31,72	0
40,36	0
40,18	0
24,68	0
56,49	0
48,40	0
64,48	1
56,96	0
17,38	1
41,19	0
48,85	0
53,62	0
41,02	0
28,99	0
47,46	0
41,59	0
33,83	0
46,30	0
54,41	1
38,28	0
30,26	0
45,03	0
35,77	0
55,07	0
21,60	0
21,94	0
51,98	0
45,55	0
22,56	0
26,54	0
35,65	0
45,34	0
54,65	1
35,32	0
30,16	0
56,01	0
55,83	0
25,86	0
28,41	0
49,23	0
52,81	0
27,44	0
45,87	0
42,06	0
59,19	0
35,54	0
46,93	0
46,38	0
58,63	0
54,77	1
60,85	0
28,40	0
66,65	0

59,94	0
26,15	0
43,32	0
67,03	0
29,34	0
60,19	0
29,82	0
22,45	0
57,91	0
98,38	0
72,48	0
37,55	1
30,69	1
26,96	0
56,50	0
39,55	1
35,51	0
73,78	0
53,88	0
32,78	0
40,43	0
20,02	0
52,26	0
31,25	0
47,46	0
48,46	0
52,10	0
26,92	1
30,04	0
41,90	0
40,87	0
47,08	0
27,98	0
33,63	0
44,99	0
22,25	1
35,43	1
27,64	0
38,02	0
59,94	0
21,70	1
51,79	0
35,84	0
39,41	0
33,66	0
34,17	0
39,43	0
67,01	0
50,62	0
37,89	0
22,99	0
45,83	0
30,44	0
63,03	0
54,22	0
40,91	0
41,23	1
8,71	2
25,17	0

23,82	0
36,15	0
36,12	0
35,62	2
51,81	0
27,19	0
33,72	1
35,57	0
25,23	0
25,70	0
